# Assessment of the potential of *Vachellia seyal* and *Prosopis chilensis* for the reclamation of saline soil lands in the peanut basin production of Senegal

**DOI:** 10.3389/fpls.2022.1001895

**Published:** 2022-12-09

**Authors:** Mansour Thiao, Godar Sene, Moustapha Ndiaye, El Hadji Samba Ndao Sylla

**Affiliations:** ^1^ Laboratoire Commun de Microbiologie (LCM) Institut de Recherche pour le Developpement/Institut Sénégalais de Recherche Agricole/Université Cheikh Anta Diop (IRD/ISRA/UCAD), Département de Biologie végétale, Faculté des Sciences et Techniques, Université Cheikh Anta Diop de Dakar, Dakar, Senegal; ^2^ Laboratoire de Botanique, Département de Biologie végétale, Faculté des Sciences et Techniques, Université Cheikh Anta Diop de Dakar, Dakar, Senegal

**Keywords:** *Vachellia seyal*, *Prosopis chilensis*, herbaceous, arbuscular mycorrhizal fungi, salinity, soil fertility

## Abstract

Soil properties and microbial activities are indicators that shape plant communities and evolution. We aimed to determine the interdependency between trees, belowground herbaceous plants, soil characteristics, and arbuscular mycorrhizal communities. *Vachellia seyal* and *Prosopis chilensis* and their associated herb layers were targeted. Soils sampled beneath the trees and outside the canopies were subjected to physicochemical and microbial characterization. Randomly collected living roots of trees and dominant herbs were checked for arbuscular mycorrhizal colonization. A tree seedlings nursery was conducted using black bags filled with the following substrates: natural soil 100%, soil mixed with leaf tree plants (LTPs) as organic matter at 10%, soil mixed with LTP at 20%, soil mixed with LTP at 30%, and soil mixed with LTP at 50%. As a result, the presence of trees improves both herb richness and diversity. Soil mycorrhizal inoculum potentials are higher beneath *V. seyal* than *P. chilensis* and decreased significantly with increasing distance from trees. The soil MIP decreased with increasing organic matter content for both tree species but was more pronounced for *P. chilensis*. Soil salinity is lower beneath *V. seyal* and higher under *P. chilensis* and outside the canopies. Soil fertility parameters such as carbon, nitrogen, and available phosphorus are higher beneath the trees and then decreased as the distance to the trees increases. We conclude that microbial communities, soil properties, and herb richness and diversity increased beneath the trees but decreased with increasing distance from the trees. This effect is tree species-dependent as *P. chilensis* increased soil salinity and decreased the belowground density of herbs.

## Introduction

Land salinization is affecting more and more lands around the world (Sène, 2012; Fall, 2016). In Senegal, the area covered by salinization was estimated at 1,200,000 ha (Sieverding, 1991). This phenomenon of salinization is distributed in Senegal in the coastal areas and the catchment areas of the Senegal, Sine, Saloum, and Casamance rivers, thus affecting a large part of the Senegalese groundnut basin. Most crops such as cereals are sensitive to salt (Evelin et al., 2009); thus, vegetation cover and agricultural production decrease accordingly. The main effects of salinity responsible for low plant productivity are growth suppression due to generally low osmotic potential during germination, emergence, and seedling growth (Miller and Jastrow, 2000) and growth suppression due to specific ion toxicity. In Senegal, in the southern part of the Fatick region, the land affected by salinization is in an ecosystem where the tree species *Vachellia seyal* and *Prosopis chilensis* are well represented and are symbiotic partners of soil microorganisms (Faye et al., 2019). Arbuscular mycorrhizal fungi (AMF) are widely present in saline soils (Aliasgharzadeh et al., 2001), but the presence of salt in soil can affect the establishment of a functional AMF symbiosis (Liu et al., 2018). Plant root colonization by AMF can also be reduced in the presence of sodium chloride (He et al., 2007). AMF symbiosis can mitigate the effects of salt stress on plants (Aroca et al., 2013). The telluric microflora, through its abundance and diversity, can play a fundamental role in restoring and maintaining soil fertility, protecting plants against telluric pathogens, as well as plant tolerance to environmental stresses and plant hydromineral nutrition. The AMF represented in these soil microbial communities are involved in the mobilization of phosphorus (Yang et al., 2014) and water (van der Heijden et al., 1998), respectively. They thus contribute to plant adaptation in poor soils and in soils subjected to environmental stress. Their abundance and specific diversity in soils could be synonymous with ecological behavior in response to abiotic and biotic factors. There are also other organisms that perform biological functions in the functioning of the soil ecosystem. For several decades, in the Sahelian zones, climatic disturbances associated with anthropic pressure have led to the disappearance of a large part of the vegetation cover. Climate change, the increase in the area of land affected by drought and salinization, anthropogenic pressure on land, and the resettlement of displaced populations have led to significant changes in ecosystems. This is reflected in places by a reduction in arable land, a decrease in vegetation cover, and the abandonment by populations of land whose use is expensive and difficult to achieve. Attempts at reforestation have already been undertaken with monospecific tree plantations (Smith and Read, 1997). Knowledge of the soil microflora associated directly or indirectly with trees in plantations and natural stands is an important link in the management of soil fertility and ecosystem stability. AMF are microsymbionts whose activities can have impacts on the structure and evolution of plant communities. In order to achieve greater efficiency in soil fertility management, it is therefore important to exploit all the potentialities offered by soil AMF in relation to plant formations. From this point of view, the analysis of the structure of plant formations whose components are interrelated with the AMF becomes an important indicator. For this purpose, phytosociology provides effective study tools that give useful information in the diagnosis and interpretation of results in the case of studying the evolution and stability of vegetation. The information collected from the studies of vegetation and its interactions with soil microorganisms could allow the identification of the most important factors in the evolution and stability of vegetation.

## Material and methods

### Phytosociological survey

#### Description of the study area, device model, and phytosociological survey method used

The area of interest for this study is located in Sadioga (GPS coordinates *X* = 344398, *Y* = 1579053) in Senegal, about 77 km south of Fatick City. The average annual temperature is 37°C. Like Senegalese regions, there are about three rainy months (July, August, and September), with a maximum of rainfall in August. The floristic study was carried out at the edge of the tannas (salt-affected soils near the salted lake), during the rainy season, in a homogeneous landscape with a predominance of two tree species *V. seyal* and *P. chilensis* in the tree layer. The trees under which the surveys were carried out have areas where their canopies overlap. Thus, five devices representing five trees of *V. seyal* and five trees of *P. chilensis* (equidistant at 20 ± 3 m) satisfying this choice were located. Phytosociological surveys, with average diameters of 9.3 m ± 10 cm, were carried out under the canopies of *V. seyal* trees and *P. chilensis* trees and outside the canopies of the trees. To do this, stakes were set up at the edge of the branches around the trees, and then a rope was used to connect the stakes in order to delimit the area where the encountered herbaceous species were recorded. Herbaceous plants were then identified at the site by observing their respective vegetative apparatus (stems, leaves, and flowers) and using identification flora tools (Berhaut, 1971; Noba and Ba, 1992; Mbaye et al., 2001; Noba, 2002; Noba et al., 2004; Sarr et al., 2007; Bassène, 2008; Ngom et al., 2016). Circular phytosociological surveys with a diameter of 9.3 m ± 10 cm were also carried out outside the canopies at 20 m far from the trunks of trees. For each survey, every herbaceous specie was listed and then assigned a semiquantitative coefficient of abundance–dominance and a coefficient of sociability/aggregation as well as a presence/absence coefficient. The abundance/dominance coefficients assigned to the species encountered at the field were transformed into semiquantitative abundance/dominance codes (AD code) according to the Braun–Blanquet scale and into quantitative codes (AD num) as shown in **Table 1** (Gillet, 2000).

**Table 1:** Correspondence between the abundance–dominance code (AD), quantitative abundance–dominance coefficient (AD num), and average, minimum, and maximum cover (Gillet, 2000).

AD code	AD num	Rec.moy	Rec.min	Rec.max
5	5	90	75	100
4	4	57	50	75
3	3	32	25	50
2	2	14	5	25
1	1	3	1	5
+	0.5	0.3	0.1	1
r	0.1	0.03	0	0.1

#### Analyses of survey similarities and association between species

The similarity between surveys was estimated by calculating Jacquard’s similarity coefficient (CS_J_). The number of common species between surveys R1 (beneath *V. seyal*) and R2 (beneath *P. chilensis*), between R1 and R3 (outside the canopies of the tree), and between R2 and R3 was determined. The formula CS_J_ = a/(a + b + c), where “a” is the number of species common to both surveys, “b” is the number of species specific to the first survey, and “c” is the number of species specific to the second survey, was applied. Species associations were determined by calculating Jacquard’s association coefficients (CA_J_). The formula CA_J_ = a/(a + b + c), where “a” is the number of surveys in which the two species being compared are present together, “b” is the number of surveys in which the first species is present alone, and “c” is the number of surveys in which the second species is present alone, was applied.

#### Living root sampling

Living root samples were collected *in situ* at the site from the trees of both *V. seyal* and *P. chilensis* species and from herbaceous plants found under the trees and outside of their canopies. The collected roots were then placed in labeled tubes containing ethanol (70%) and placed in a temperature controller. On arrival at the laboratory, the roots were thoroughly rinsed with sterile demineralized water, soaked in 70% ethanol, and stored at 4°C until analysis.

### Soil sampling and analysis

Soil samples were taken at the edge of the tannas under the canopies of *V. seyal* and *P. chilensis* trees at 0 m near the trunks and outside the canopies of trees at 20 m far from the trunks. For each of the soil samples, four replicates were taken, which corresponds to a total of 12 samples for each of the five devices. At each sampling, the surface of soil containing debris was cleared, and soil was collected from the depth horizon 0–25 cm. Soil analyses were carried out by the Soil and Plant Analyses Laboratory of the Centre of Agricultural Research (CRA) at Saint-Louis (Senegal). For each soil sample, particle size, electrical conductivity (EC), carbon-to-nitrogen ratio (C/N), organic matter (OM), nitrogen (N), total phosphorus(P_2_O_5_), calcium (Ca^2+^), available phosphorus (P ass), sodium (Na^+^), potassium (K^+^) and magnesium (Mg^2+^) contents, cationic exchange capacity (CEC), water pH, and KCl pH were analyzed using standard analytical methods.

### Mycorrhizal inoculum potentials of soil

The mycorrhizal inoculum potential (MIP) of soils was estimated by measuring the AMF colonization of living roots, the most probable number (MPN) of propagules capable to generate mycorrhizae as described by Plenchette et al. (1989), the root AMF colonization rate in soil collected beneath the trees and outside of canopies, and the AMF colonization of roots in the presence of several rates of organic matter (OM).

#### Living root AMF colonization

For natural mycorrhization assessment, living root samples were collected *in situ* from the trees of both *V. seyal* and *P. chilensis* species and from dominant herbaceous plants found under and outside the canopies of the tree species. Collected roots were then placed in labeled tubes containing ethanol (70%) and placed in a temperature controller. On arrival at the laboratory, the roots were thoroughly rinsed with sterile demineralized water, soaked in 70% ethanol, and stored at 4°C until analysis. The roots were then processed as below for mycorrhization rate determination. A comparison test was applied to define groups based on species frequencies of mycorrhization and intensities of mycorrhization rates.

##### Root thinning

Roots were first treated with 10% KOH in a water bath (at 100°C) for 45 min for herbaceous plants and 60 min for trees in order to empty their cellular contents (Phillips and Hayman, 1970). This treatment was repeated 30 min later, and residual KOH was removed from the roots by a series of rinses with tap water. The roots were then placed in bleach diluted 8-fold for 10 min to complete the clearing and rinsed again several times with tap water. They were then placed in 5% hydrochloric acid for 10 min to neutralize the bleach.

##### Root staining

The thinned roots were then stained by dipping in 0.05% trypan blue in a solution with an equal volume of lactic acid, glycerol, and distilled water (Phillips and Hayman, 1970). The mixture was then placed in a water bath at 100°C for 60 min. After this staining step, the excess dye was removed by rinsing with sterilized demineralized water, and the roots were stored at 4°C in the last wash water until they were observed.

##### Observation of the roots

For each sample, root fragments of about 1 cm were placed between the slide and the coverslip, crushed in glycerol, and observed under a microscope. The observation of hyphae, vesicles, or arbuscules in the roots was used to estimate the levels of colonization (endomycorrhization) of the root samples. The estimation of root colonization by AMF was done using the method of Trouvelot et al. (1986). The latter established a scoring system to estimate the proportion of root cortex colonized by AMF based on six classes and the presence of arbuscules (in the mycorrhizal part) based on four classes. These different classes are described in the diagram below.

##### Estimation of root mycorrhization

The frequency and intensity of mycorrhization were calculated according to the following formulas:

Frequency of mycorrhization of the root system noted F % = (number of mycorrhizal fragments)

F% = (number of mycorrhized fragments/total number of observed fragments) × 100

Mycorrhization intensity of the system noted I% = (95n5+)

I% = (95n5 + 70n4 + 30n3 + 5n2 + n1)/total number of fragments observed

Where n5 = number of fragments scored 5, n4 = number of fragments scored 4, n3 = number of fragments scored 3, n2 = number of fragments scored 2, and n1 = number of fragments scored 1.

#### Most probable number of AMF community

The capacity of soils to support mycorrhization from propagules (spores, infected root fragments, and mycelium) was estimated by the MPN method described by Plenchette et al. (1989). Maize (*Zea mays*), which is a highly mycotrophic species (Wang et al., 2008; Abdelilah et al., 2017), was used as an endophytic plant. For each sample, sieved dry soil (<2 mm) was autoclaved at 140°C for 45 min. A series of 1/10th dilutions from 10^−1^ to 10^−6^ was performed with five replicates per dilution level. Dilutions were made by homogenizing each sample with different proportions of non-sterile and sterilized soil. Maize seeds were disinfected in bleach (90%) diluted by half for 3 min and then in 70% ethanol for 3 min. They were then rinsed several times with sterile demineralized water and soaked in sterile demineralized water for 3 h. After soaking, they were sown in pots at a rate of two seeds per pot. After germination, the seedlings were removed to one plant and watered as needed for 6 weeks. After 6 weeks of cultivation, the maize plants were harvested and the roots carefully rinsed with sterilized demineralized water and kept in cold storage (4°C) in 70% alcohol. The harvested roots were then processed as described above, and the number of propagules capable of generating mycorrhizae per gram of soil was deduced from the reading (Sieverding, 1991) with a probability of 95% according to the table of Cochran (1950).

#### Symbiotic root colonization by AMF community in soil collected beneath the trees and outside of canopies

For experimental determination of mycorrhization rate, maize seeds were used for germination of cultivation. They were then sown in the soils. For each soil sampled, a replicate of five plants was applied. After 8 weeks of cultivation, the plants were harvested and the root systems were used to estimate mycorrhization rates. The frequency (*F*) of colonization was determined by noting the presence or absence of propagules (hyphae, vesicles, or arbuscules) in each root fragment. Each observation was scored + (presence of propagules) or − (absence of propagules).

##### Soil mycorrhizal infection potential in the presence of organic matter

A randomized complete block design using bulk soil samples was conducted in a nursery using *V. seyal* and *P. chilensis* seedlings cultivated in black polythene bags (8 × 13 cm with 200 ml capacity) filled with five substrate types: natural soil 100% (substrate A), soil (90%) mixed with leaf tree plants (LTPs) as organic matter at 10% (substrate B), soil (80%) mixed with LTP at 20% (substrate C), soil (70%) mixed with LTP at 30% (substrate D), and soil (50%) mixed with LTP at 50% (substrate E). These mixtures were composted for 1 month and then the nursery of *V. seyal* was set on a substrate of *V. seyal* organic matter and the nursery of *P. chilensis* was set on a substrate containing *P. chilensis* organic matter. Replicates of five were made for each level of organic matter and for all soils. Seeds of *V. seyal* and *P. chilensis* were then treated and sown in pots. The seedlings were grown for 3 months in a greenhouse. At the end of the 3 months of cultivation, mycorrhization rates were assessed as described above.

### Statistical analysis

Phytosociological data were analyzed using the phytosociological tool presence–absence, association index, and abundance–dominance indexes and by calculating Jacquard’s coefficients similarity and Jacquard’s association indexes. Other data were analyzed with the *RStudio* application using Kruskal and ANOVA tests.

## Results

### Phytosociological survey

#### Phytosociological and similarity analyses and species associations

The results of floristic and ecological analyses of herbaceous species are shown in **Table 2** (presence–absence), **Table 3** (association index), and **Table 4** (abundance–dominance indexes). The number of species found in all surveys is 29. Five species were found beneath the trees and were not found outside the canopies. The number of common species between surveys under canopies of *V. seyal* and *P. chilensis* is 16. The number of species found only beneath the trees of *V. seyal* surveys is 9. The number of species found only beneath the trees of *P. chilensis* surveys is 3. Jacquard’s coefficient of similarity CS_J_ between the *V*. *seyal* and *P. chilensis* surveys is equal to: $CSj=\frac{16}{16+9+3}$ = 0.57.

**Table 2:** The presence–absence of herbaceous species beneath *Vachellia seyal* and *Prosopis chilensis*, and outside their canopies.

Species	R1	R2	R3	Fr
*Melhania denhamii* R. Br.	1	1	1	3
*Eragrostis tremula* Steud.	1	1	1	3
*Desmodium hirtum* Guill. & Perr.	1	1	1	3
*Ipomoea kotschyana* Hochst. ex Choisy	1	1	1	3
*Cyperus rotundus* L.	1	1	1	3
*Zornia glochidiata* Reichb. ex DC	1	1	1	3
*Fimbristylis ferruginea* (L.) Vahl	1	1	1	3
*Oldenlandia corymbosa* L.	1	1	1	3
*Blutaparon vermiculare* (L.) Mears	1	1	1	3
*Dactyloctenium aegyptium* (L.) P. Beauv.	1	1	1	3
*Enteropogon prieuri* (Kunth) Clayton.	1	1	1	3
*Aeschynomene indica* L.	1	1	0	2
*Paspalum dilatatum* Poir.	1	1	0	2
*Waltheria indica* L.	1	1	0	2
*Leptothrium senegalense* (Kunth)	1	1	0	2
*Brachiaria ramosa* (L.) Stapf	1	1	0	2
*Bulbostylis hispidula* (Vahl) Haines	1	0	1	2
*Cyperus esculentus* L.	1	0	1	2
*Alysicarpus ovalifolius* (Schumach.) Leonard	1	0	1	2
*Mitracarpus villosus* (Sw.) DC.	0	1	1	2
*Urginea indica* (Roxb.) Kunth.	1	0	0	1
*Panicum laetum* Kunth	1	0	0	1
*Kohautia grandiflora* DC.	1	0	0	1
*Blepharis maderaspatensis* (L.) B. Heyne ex Roth	1	0	0	1
*Peristrophe bicalyculata* (Retz.) Nees	1	0	0	1
*Corchorus tridens* L.	1	0	0	1
*Blainvillea gayana* auct.	0	1	0	1
*Achyrantes argentea* Lam.	0	1	0	1
*Senna obtusifolia* (L.) H.S. Irwin & Barneby	0	0	1	1
Number of species	25	19	16	29

1, presence; 0, absence; Fr, frequency; R1: survey beneath V. seyal, R2: survey beneath P. chilensis, R3: survey beneath outside the canopies of trees.

**Table 3:** Association coefficient of *Vachellia seyal* and of *Prosopis chilensis* vs. herbaceous species.

Tree species	Associated herbaceous species	Association coefficient
*V. seyal*	*M. denhamii*	0.33
*V. seyal*	*A. indica*	0.66
*V. seyal*	*E. tremula*	0.33
*V. seyal*	*D. hirtum*	0.33
*V. seyal*	*I. kotschyana*	0.33
*V. seyal*	*C. rotundus*	0.33
*P. chilensis*	*C. rotundus*	0.33
*P. chilensis*	*B. vermiculare*	0.33
*P. chilensis*	*B. gayana*	1
*P. chilensis*	*I. kotschyana*	0.33
*P. chilensis*	*A. indica*	0.5
*P. chilensis*	*D. hirtum*	0.33
*P. chilensis*	*E. prieuri*	0.33
*P. chilensis*	*E. tremula*	0.33

**Table 4:** Crude floristic table beneath *Vachellia seyal* and *Prosopis chilensis* trees and outside their canopies.

Encountered species	R1: beneath *V. seyal*	R2: beneath *P. chilensis*	R3: outside the canopies
*Eragrostis tremula* Steud.	3	1	3
*Melhania denhamii* R. Br.	3	+	r
*Desmodium hirtum* Guill. & Perr.	2	1	r
*Aeschynomene indica* L.	1	1	
*Bulbostylis hispidula* (Vahl) Haines	1		+
*Cyperus rotundus* L.	1	3	+
*Ipomoea kotschyana* Hochst. ex Choisy	1	1	1
*Zornia glochidiata* Reichb. ex DC	1	r	1
*Achyrantes argentea* Lam		+	
*Alysicarpus ovalifolius* (Schumach.) Leonard	R		r
*Blainvillea gayana* auct.		1	
*Blepharis maderaspatensis* (L.) B. Heyne ex Roth	R		
*Blutaparon vermiculare* (L.) Mears	R	1	+
*Corchorus tridens* L.	R		
*Cyperus esculentus* L.	+		+
*Dactyloctenium aegyptium* (L.) P. Beauv.	R	r	r
*Enteropogon prieuri* (Kunth) Clayton.	R	1	3
*Fimbristylis ferruginea* (L.) Vahl	+	+	+
*Kohautia grandiflora* DC.	+		
*Mitracarpus villosus* (Sw.) DC.		r	+
*Oldenlandia corymbosa* L.	+	+	r
*Panicum laetum* Kunth	+		
*Paspalum dilatatum* Poir.	+	r	
*Peristrophe bicalyculata* (Retz.) Nees	R		
*Leptothrium senegalense* (Kunth)	R	r	
*Senna obtusifolia*			1
*Urginea indica* (Roxb.) Kunth.	+		
*Waltheria indica* L.	R	r	
*Brachiaria ramosa* (L.) Stapf	R	r	

Rows: species; columns: surveys (R1, R2, R3); numbers, signs, and letters: index of abundance–dominance (Braun-Blanquet, 1964).

The number of common species between surveys beneath the trees of *V. seyal* and outside tree canopies is 14. The number of species found only beneath the trees of *V. seyal* surveys is 11. The number of species found only outside the canopies is 2. Jacquard’s similarity coefficient CS_J_ between surveys beneath the trees of *V. seyal* and outside the canopies is equal to: $CSj=\frac{14}{14+11+2}$ = 0.52.

The number of common species between the surveys beneath the trees of *P. chilensis* and outside the canopies is equal to 12. The number of species found only beneath the trees of *P. chilensis* surveys is 7. The number of species found only out of the tree canopies is 4. Jacquard’s similarity coefficient CS_J_ between beneath the trees of *P. chilensis* and outside of tree canopies surveys is as follows: $CSj=\frac{12}{12+7+4}$ = 0.52.

The coefficients of association (CAs) calculated (**Table 3**) between *V. seyal* and the five herbaceous species that have the highest cover beneath the trees of *V. seyal* are CA_(_ *_V.seyal_vs_M.denhamii_*_)_ = 0.33, CA_(_ *_V.seyal_vs_E.tremula_*_)_ = 0.33, CA_(_ *_V.seyal_vs_D.hirtum_*_)_ = 0.33, CA_(_ *_V.seyal_vs_I.kotschyana_*_)_ = 0.33, and CA_(_ *_V.seyal_vs_C.rotundus_*_)_ = 0.33.

The calculated CAs (**Table 3**) between *P. chilensis* and the eight herbaceous species that have the highest cover beneath the trees of *P. chilensis* are CA_(_ *_P.chilensis_*__vs__ *_C.rotundus_*_)_ = 0.33, CA_(P_ *_.chilensis_*__vs__ *_B.vermiculare_*_)_ = 0.33, CA_(_ *_P.chilensis_*__vs_ *__B.gayana_*_)_ = 1, CA_(_ *_P.chilensis_*__vs__ *_I.kotschyana_*_)_ = 0.33, CA_(_ *_P. chilensis_*__vs__ *_A.indica_*_) =_ 0.5, CA_(_ *_P.chilensis_vs_D.hirtum_*_)_ = 0.33, CA_(_ *_P.chilensis_*__vs__ *_E.prieuri_*_)_ = 0.33, and CA_(_ *_P.chilensis_*__vs__ *_E.tremula_*_)_ = 0.33.

The abundance–dominance coefficients of the herbaceous species are shown in **Table 4**. Beneath the trees of *V. seyal*, *Melhania denhamii* R. Br. and *Eragrostis tremula* Steud. have the highest values (3) of AD code. After these two species, *Desmodium hirtum* Guill. & Perr. has an AD code of 2. The species *Bulbostylis hispidula* (Vahl) Haines, *Cyperus rotundus* L., *Ipomoea kotschyana* Hochst. ex Choisy, *Aeschynomene indica* L., and *Zornia glochidiata* Reichb. ex DC have an AD code of 1. The other species represented beneath the trees of *V. seyal* have AD codes of + or r. Beneath the trees of *P. chilensis*, only *C. rotundus* has an AD code of 3. The species *E. tremula*, *D. hirtum*, *I. kotschyana*, *Blainvillea gayana*, *Blutaparon vermiculare*, and *Enteropogon prieuri* have AD codes of 1. The other species found beneath the trees of *P. chilensis* have AD codes of + or r. Outside the canopies of the trees, only *E. tremula* has an AD code of 3. The species *I. kotschyana*, *Z. glochidiata*, and *Senna obtusifolia* have AD codes of 1. The other species found outside the canopies of the trees have AD codes of + or r.

In **Table 4**, the coefficients 0 and 1 indicate if species were found (1 = presence) or not (0 = absence), respectively. The values 3, 2, and 1 in the last column (Fr) indicate how many times the corresponding species were found in the surveys (absolute species frequencies). The values in the last row indicate the number of species per survey, and the last number in this row represents the total number of species recorded (observed species richness). The average number of species per survey is 20 (total number of species for all surveys divided by the number of surveys) with a variance of 1.45 (total number of species 29 divided by the average number of species 20). The analysis in **Tables 2** and **4** shows that the species richness (or floristic richness) varies between 16 and 25 and there are 29 species for all the surveys. The average species richness is 20 with a variance of 1.45. It is higher beneath the trees than outside the crowns. Under the trees, it is higher beneath *V. seyal* than beneath *P. chilensis*.

The herbaceous species *M. denhamii*, *E. tremula*, *D. hirtum*, *I. kotschyana*, *C. rotundus*, *Z. glochidiata*, *Fimbristylis ferruginea*, *Oldenlandia corymbosa*, *B. vermiculare*, *Dactyloctenium aegyptium*, and *E. prieuri* are associated with *V. seyal*, *P. chilensis*, and the area outside the canopies of the trees. These are the most frequent species when considering all the surveys with a relative frequency of 3. They are the common species. Among them, *E. tremula* has the highest abundance–dominance coefficient (AD code) with a value of 3 both beneath the trees of *V. seyal* and outside the canopies of the trees, corresponding to a coverage of 25% to 50% of the survey area. In contrast, its individuals are scattered with an AD code of 1 beneath the trees of *P. chilensis*. *Melhania denhamii* has an AD code value of 3 beneath the trees of *V. seyal*, but beneath the trees of *P. chilensis*, it has few individuals with low cover and rare individuals outside the tree canopies. *Enteropogon prieuri* has an AD code of 3 outside the canopies of the trees. In contrast, its individuals are rare beneath the trees of *V.* seyal and scattered beneath the trees of *P. chilensis*. *Desmodium hirtum* has an AD code of 2 corresponding to a cover of 25% to 50% of the surface beneath the trees of *V. seyal*. In contrast, beneath the trees of *P. chilensis* and outside the canopies of the trees, its individuals are respectively scattered and rare. For *C. rotundus*, it presents an AD code of 3 (25% to 50% cover) beneath the trees of *P. chilensis*, but its individuals are scattered with a low cover beneath the trees of *V. seyal* and are rare outside the tree canopy. The other species are globally present with scattered and rare individuals beneath the trees and outside the canopies of trees.

The herbaceous species *A. indica*, *Paspalum dilatatum*, *Waltheria indica*, *Leptothrium senegalense*, and *Brachiaria ramosa* are related to *V. seyal* and *P. chilensis*. They have a relative frequency of 2 and have not been found outside the tree canopies. They belong to the rare and infrequent species. Their individuals are scattered and rare with AD codes of 1, +, and r. The other species below have their individuals either scattered or rare depending on the case. The herbaceous species *B. hispidula*, *Cyperus esculentus*, and *Alysicarpus ovalifolius* are associated with *V. seyal* and the area outside the tree canopy with a relative frequency of 2. They belong to the infrequent rare species. They have not been found beneath the trees of *P. chilensis*. Their individuals are scattered and rare with low cover. The species *Mitracarpus villosus* is associated with *P. chilensis* and the area outside the tree canopy with a relative frequency of 2. It is a rare and infrequent species. It presents a few individuals with a low cover outside the canopy and rare individuals beneath the trees of *P. chilensis*. It was not found beneath the trees of *V. seyal*. The herbs *Urginea indica*, *Panicum laetum*, *Kohautia grandiflora*, *Blepharis maderaspatensis*, *Peristrophe bicalyculata*, and *Corchorus tridens* are only associated with *V. seyal* with a relative frequency of 1. The first three mentioned have few individuals with very low cover and the last three have rare individuals. They have not been found beneath the trees of *P. chilensis* and outside the canopies of the trees. They are among the unique species encountered only once. The same is true for *B. gayana* and *Achyrantes argentea* which are related to *P. chilensis* with a relative frequency of 1, with respectively scattered and few individuals with very low cover and which were not encountered beneath the trees of *V. seyal* and in the area outside the tree canopies. *Senna obtusifolia* is related to the area outside the canopies with a relative frequency of 1 and is one of the single species with scattered individuals. It was not found beneath the tree species.

#### Structure and coverage percentages of the herbaceous flora

##### Structure of the herbaceous flora beneath *Vachellia seyal*

The structure of the herbaceous flora beneath *V. seyal* is represented in **Table 5**. Beneath the canopies of the trees of *V. seyal*, dicotyledons are represented by six families, 13 genera, and 13 species. Monocotyledons are represented by three families, 11 genera, and 12 species. The proportions of families, genera, and species in these two classes are 66.67% vs. 33.33%, 54.17% vs. 45.83%, and 52% vs. 48%, respectively. Thus, a total of nine families, 23 genera, and 25 species were recorded. Twenty-five herbaceous species are found beneath the trees of *V. seyal*. In the area up to a diameter of about 2.2 m from the trunk, *M. denhamii* has a greater number of individuals, and its sociability coefficient reaches 5. The density of its individuals started to decrease, and individuals of *E. tremula* became more abundant from 2.2 to 2.7 m at the periphery of the survey area. In this part area the trees of *V. seyal*, the sociability coefficient of *M. denhamii* decreases to 3. In the area outside the *V. seyal* canopies, the sociability coefficient of *M. denhamii* comes down to 1. In the area beneath the trees of the *V. seyal* canopy close to the periphery, the sociability coefficient of *E. tremula* is 2. Beyond this and in the uncovered area, the sociability coefficient of *E. tremula* is 3, and its individuals occupy most of the herbaceous cover, with individuals forming dense groups in some places. After *M. denhamii*, *D. hirtum* had more individuals in the vicinity of the *V. seyal* trunk with a sociability coefficient of 3. The other species recorded beneath the trees of *V. seyal* had a sociability coefficient of 1.

**Table 5:** Structure of the herbaceous flora.

		Families			Genres			Species	
		Number		Frequencies	Number		Frequencies	Number	Frequencies
Beneath the trees of *V. seyal*	Dicotyledons	6		66.67%	13		54.17%	13	52%
	Monocotyledons	3		33.33%	11		45.83%	12	48%
Total		9		100%	24		100%	25	100%
		Families			Genres			Species	
		Number		Frequencies	Number	Frequencies		Number	Frequencies
Beneath the trees of *P. chilensis*	Dicotyledons	6		75%	11	57.90%		11	57.90%
	Monocotyledons	2		25%	8	42.10%		8	42.10%
Total		8		100%	19	100%		19	100%
		Families			Genres			Species	
		Number	Frequencies		Number	Frequencies		Number	Frequencies
20 m from the canopies of trees	Dicotyledons	5	71.43%		9	60%		9	56.25%
	Monocotyledons	2	28.57%		6	40%		7	43.75%
Total		7	100%		15	100%		16	100%

##### Structure of the flora beneath *Prosopis chilensis*

The structure of the herbaceous flora beneath *P. chilensis* is represented in **Table 5**. Beneath the trees of *P. chilensis*, dicotyledons are represented by six families, 11 genera, and 11 species. Monocotyledons are represented by two families, eight genera, and eight species. The proportions in terms of families, genera, and species between the two classes are 75% vs. 25%, 57.90% vs. 42.10%, and 57.90% vs. 42.10%, respectively. Thus, eight families, 19 genera, and 19 species were recorded. Nineteen (19) herbaceous species were found beneath the trees of *P. chilensis*. The area around the trunk up to about 1.5 to 2 m is sparse. The few individuals present are widely spaced. In the area toward the periphery, the density of individuals increases moderately and remains low with a predominance of *C. rotundus* which is the species with the most individuals. Its sociability coefficient reaches 3. The sociability coefficient of the other species present in a very sparse manner is 1. The area beneath the trees of *P. chilensis* has far fewer individuals compared with the area beneath the trees of *V. seyal*.

##### Structure of the flora outside the canopies of the trees

The structure of the herbaceous flora outside the canopies of *V. seyal* and *P. chilensis* is represented in **Table 5**. Outside the canopies of the trees, in the herbaceous flora, dicotyledons are present with five families, nine genera, and nine species. Monocotyledons are represented by two families, six genera, and seven species. The proportions in terms of families, genera, and species between the two classes are 71.43% vs. 28.57%, 60% vs. 40%, and 56.25% vs. 43.75%, respectively. Thus, seven families, 15 genera, and 16 species were recorded. The number of herbaceous species found outside the canopies of the trees is 16. The two most represented species are *E. prieuri* and *E. tremula*. Their sociability coefficient is 4. *Senna obtusifolia* was only found outside the canopies of the trees. The sociability coefficient of the other species is 1.

#### Coverage percentages of herbaceous flora

The overall herbaceous coverage beneath the trees of *V. seyal* is estimated to be 95.40% (**Table 6**). The herbaceous species *B. maderaspatensis*, *C. tridens*, *P. laetum*, *P. bicalyculata*, and *U. indica* are found only beneath the trees of *V. seyal*. The average coverage of herbaceous species beneath the trees of *P. chilensis* is estimated at 54.41% (**Table 7**). *Cyperus rotundus* occupied 58.81% of the herbaceous cover beneath the trees of *P. chilensis* (32% of the 54.41%). The species *A. argentea* and *B. gayana* are recorded only beneath the trees of *P. chilensis*. The coverage of herbaceous species outside the canopies of the trees is 77.65% (**Table 8**). The two most represented species outside the canopies of the trees are *E. prieuri* and *E. tremula* which occupy 82.42% of the herbaceous coverage.

**Table 6:** Percentages of the coverage of herbaceous species beneath *Vachellia seyal*.

Families	N.G	N.E	Species	Cover means (%)
*Malvaceae* (D)	3	3	*Melhania denhamii* R. Br.	32
*Poaceae* (M)	7	7	*Eragrostis tremula* Steud.	32
*Fabaceae* (D)	4	4	*Desmodium hirtum* Guill. & Perr.	14
*Convolvulaceae* (D)	1	1	*Ipomoea kotschyana* Hochst. ex Choisy	3
*Cyperaceae* (M)	3	4	*Bulbostylis hispidula* (Vahl) Haines	3
*Cyperaceae* (M)	3	4	*Cyperus rotundus* L.	3
*Fabaceae* (D)	4	4	*Aeschynomene indica* L.	3
*Fabaceae* (D)	4	4	*Zornia glochidiata* Reichb. ex DC	3
*Asparagaceae* (M)	1	1	*Urginea indica* (Roxb.) Kunth.	0.3
*Cyperaceae* (M)	3	4	*Cyperus esculentus* L.	0.3
*Cyperaceae* (M)	3	4	*Fimbristylis ferruginea* (L.) Vahl	0.3
*Poaceae* (M)	7	7	*Panicum laetum* Kunth	0.3
*Poaceae* (M)	7	7	*Paspalum dilatatum* Poir.	0.3
*Rubiaceae* (D)	2	2	*Kohautia grandiflora* DC.	0.3
*Rubiaceae* (D)		2	*Oldenlandia corymbosa* L.	0.3
*Acanthaceae* (D)	2	2	*Blepharis maderaspatensis* (L.) B. Heyne ex Roth	0.03
*Acanthaceae* (D)	2	2	*Peristrophe bicalyculata* (Retz.) Nees	0.03
*Amaranthaceae* (D)	1	1	*Blutaparon vermiculare* (L.) Mears	0.03
*Fabaceae* (D)	4	4	*Alysicarpus ovalifolius* (Schumach.) Leonard	0.03
*Malvaceae* (D)	3	3	*Corchorus tridens* L.	0.03
*Malvaceae* (D)	3	3	*Waltheria indica* L.	0.03
*Poaceae* (M)	7	7	*Dactyloctenium aegyptium* (L.) P. Beauv.	0.03
*Poaceae* (M)	7	7	*Enteropogon prieuri* (Kunth) Clayton.	0.03
*Poaceae* (M)	7	7	*Leptothrium senegalense* (Kunth)	0.03
*Poaceae* (M)	7	7	*Brachiaria ramosa* (L.) Stapf	0.03
Total: 95.40

N.G, number of genders; N.E, number of species.

**Table 7:** Percentages of the coverage of herbaceous species beneath *Prosopis chilensis*.

Families	N.G	N.E	Species	Mean cover (%)
*Cyperaceae* (M)	2	2	*Cyperus rotundus* L.	32
*Amaranthaceae* (D)	2	2	*Blutaparon vermiculare* (L.) Mears	3
*Asteraceae* (D)	1	1	*Blainvillea gayana* auct.	3
*Convolvulaceae* (D)	1	1	*Ipomoea kotschyana* Hochst. ex Choisy	3
*Fabaceae* (D)	3	3	*Aeschynomene indica* L.	3
*Fabaceae* (D)	3	3	*Desmodium hirtum* Guill. & Perr.	3
*Poaceae* (M)	6	6	*Enteropogon prieuri* (Kunth) Clayton.	3
*Poaceae* (M)	6	6	*Eragrostis tremula* Steud.	3
*Amaranthaceae* (D)	2	2	*Achyrantes argentea* Lam.	0.3
*Cyperaceae* (M)	2	2	*Fimbristylis ferruginea* (L.) Vahl	0.3
*Malvaceae* (D)	2	2	*Melhania denhamii* R. Br.	0.3
*Rubiaceae* (D)	2	2	*Oldenlandia corymbosa* L.	0.3
*Fabaceae* (D)	3	3	*Zornia glochidiata* Reichb. ex DC	0.03
*Malvaceae* (D)	2	2	*Waltheria indica* L.	0.03
*Poaceae* (M)	6	6	*Dactyloctenium aegyptium* (L.) P. Beauv.	0.03
*Poaceae* (M)	6	6	*Brachiaria ramosa* (L.) Stapf	0.03
*Poaceae* (M)	6	6	*Paspalum dilatatum* Poir.	0.03
*Poaceae* (M)	6	6	*Leptothrium senegalense* (Kunth)	0.03
*Rubiaceae* (D)	2	2	*Mitracarpus villosus* (Sw.) DC.	0.03
Total: 54.41

N.G, number of genders; N.E, number of species.

**Table 8:** Percentages of the coverage of herbaceous species outside the canopies of *Vachellia seyal* and *Prosopis chilensis*.

Families	N.G	N.E	Species	Mean cover (%)
*Poaceae* (M)	3	3	*Enteropogon prieuri* (Kunth) Clayton.	32
*Poaceae* (M)	3	3	*Eragrostis tremula* Steud.	32
*Convolvulaceae* (D)	1	1	*Ipomoea kotschyana* Hochst. ex Choisy	3
*Cyperaceae* (M)	3	4	*Cyperus esculentus* L.	3
*Fabaceae* (D)	4	4	*Senna obtusifolia* (L.) H.S. Irwin & Barneby	3
*Fabaceae* (D)	4	4	*Zornia glochidiata* Reichb. ex DC	3
*Amaranthaceae* (D)	1	1	*Blutaparon vermiculare* (L.) Mears	0.3
*Cyperaceae* (M)	3	4	*Bulbostylis hispidula* (Vahl) Haines	0.3
*Cyperaceae* (M)	3	4	*Cyperus rotundus* L.	0.3
*Cyperaceae* (M)	3	4	*Fimbristylis ferruginea* (L.) Vahl	0.3
*Rubiaceae* (D)	2	2	*Mitracarpus villosus* (Sw.) DC.	0.3
*Fabaceae* (D)	4	4	*Alysicarpus ovalifolius* (Schumach.) Leonard	0.03
*Fabaceae* (D)	4	4	*Desmodium hirtum* Guill. & Perr.	0.03
*Malvaceae* (D)	1	1	*Melhania denhamii* R. Br.	0.03
*Poaceae* (M)	3	3	*Dactyloctenium aegyptium* (L.) P. Beauv.	0.03
*Rubiaceae* (D)	2	2	*Oldenlandia corymbosa* L.	0.03
Total: 77.65

N.G, number of genders; N.E, number of species.

### Soil analysis

#### Water pH and KCl pH

**Figure 1:**
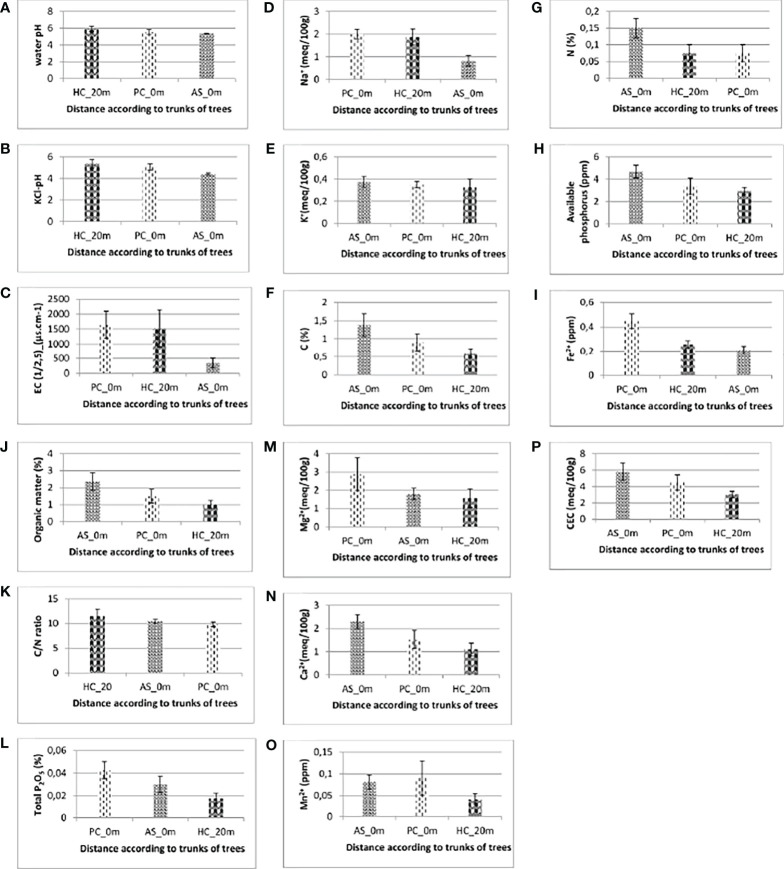
Soil water pH **(A)**, KCl-pH **(B)**, electrical conductivity **(C)**, sodium content **(D)**, potassium content **(E)**, percentages of carbon **(F)**, nitrogen content **(G)**, available phosphorus **(H)**, iron Fe2+ content **(I)**, organic matter content **(J)**, C/N ratio **(K)**, Total P2O5 content **(L)**, magnesium content **(M)**, calcium content **(N)**, manganese content **(O)** and cation exchange capacity **(P)** of soils.AS, Vachellia seyal; Pc, Prosopis chilensis; HC, out cover of trees.

The results of water pH and KCl pH are shown in **Figures 1A, B**. Soil water pH values do not show significant differences depending on whether the soil is beneath the trees of *V. seyal* and *P. chilensis* or is 20 m away from the trunks of trees. However, soil KCl pH varies significantly when comparing soils beneath the trees of *V. seyal* and *P. chilensis* and soils 20 m away from the trunks of trees. They are lower in the soils beneath the trees and are also lower for soils beneath the trees of *V. seyal* than beneath the trees of *P. chilensis*.

#### Electrical conductivity and soil sodium and potassium contents

**Figure 2:**
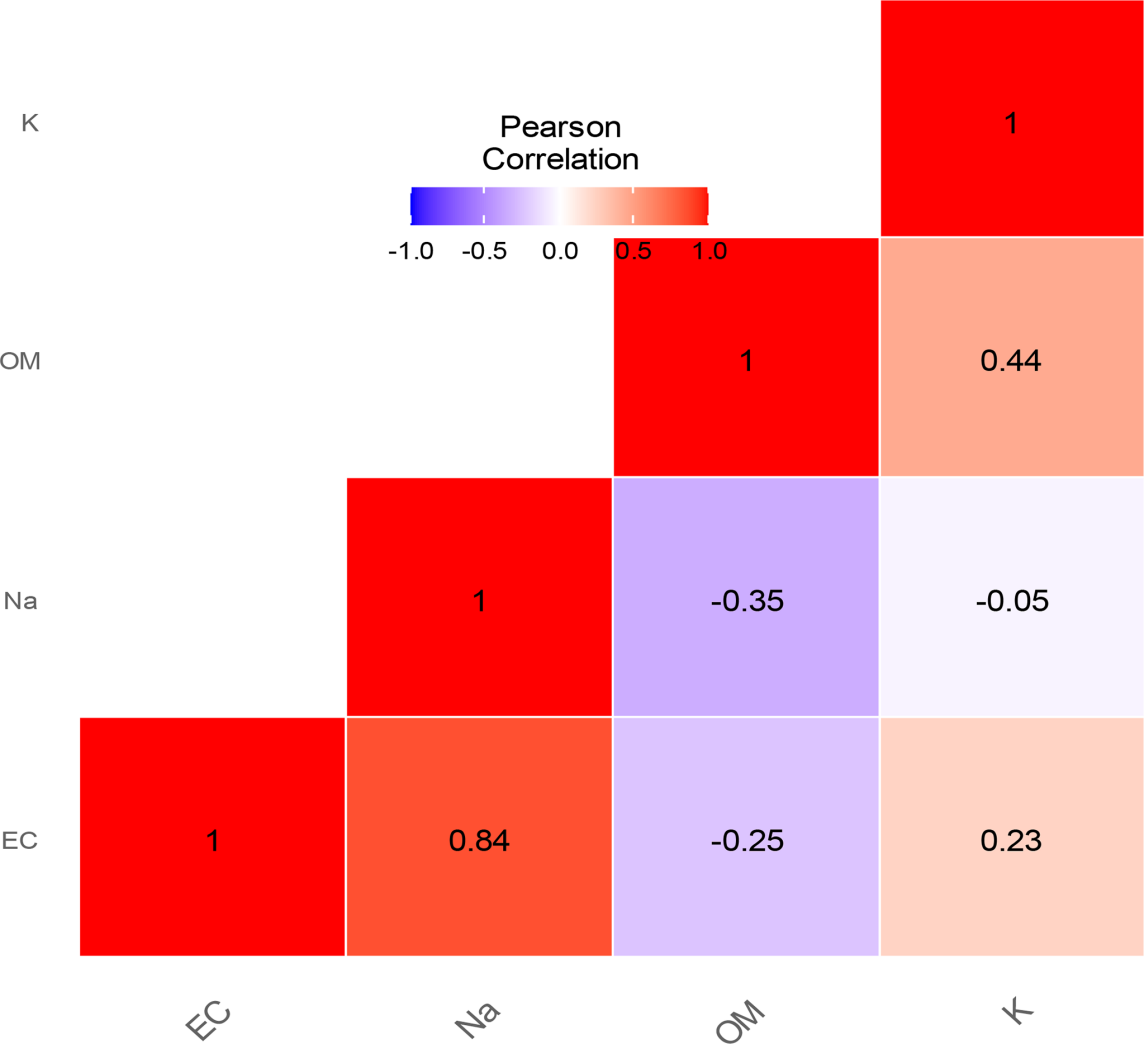
Pearson correlation between EC, Na, OM, and K.

The results of the EC and sodium content of the soils are represented, respectively, in panels **C** and **D** of **Figure 1**. The EC values varied significantly in the soil beneath *V*. *seyal* compared with the soil beneath *P. chilensis* and soil at 20 m distance from the trunks of trees. The EC is more than 4.38 times lower in the soils beneath *V. seyal* than in soils 20 m away from the trunks of trees and 4.72 times lower in the soils beneath the trees of *V. seyal* compared with the soils beneath the trees of *P. chilensis*. The values of sodium content varied significantly. The average values of sodium (Na^+^ meq/100 g) in the soils are 1.875 at 20 m distance from the trunks of trees, 2 at 0 m beneath the trees of *P. chilensis*, and 0.825 at 0 m under the trees of *V. seyal*. Thus, the amount of Na^+^ is higher in soils beneath *P. chilensis* and in soils 20 m away from the canopies of the trees. The sodium content is more than two times lower in soils under the trees of *V. seyal* compared with soils under the trees of *P. chilensis* and to soils 20 m distance from the trunks of trees. The EC and the Na^+^ content have a strong Pearson correlation link with an index of 0.84 (**Figure 2**).

#### Fertility parameters

The results of fertility parameter measurements are presented in **Figure 1**. The results of the measurements of soil potassium (K^+^) content are shown in **Figure 1E**. Statistical analysis (Kruskal, pV = 0.829 > 0.05) shows that the amount of K^+^ does not vary significantly in soils depending on the tree species and/or the distance to the trunks of the trees. In soils, the percentages of carbon (**Figure 1F**) and nitrogen (**Figure 1G**) contents and the amounts of available phosphorus (**Figure 1H**) and iron (**Figure 1I**) varied significantly depending on the distance between the soil sampling site and the trunks of trees and also depending on species (*V. seyal* or *P. chilensis*). The quantities of carbon are higher in soils at 0 m under the trees than in soils 20 m away from the trunks of trees. They are higher in soils beneath the trees of *V. seyal* than in soils beneath the trees of *P. chilensis*. The average percentage values of carbon are 0.6 at 20 m distance from the trunks of trees, 0.9 at 0 m beneath the trees of *P. chilensis*, and 1.375 at 0 m in soils beneath the trees of *V. seyal*. The percentage values of nitrogen measured are twice higher (0.15%) for soils at 0 m beneath the trees of *V. seyal* compared with soils at 0 m beneath the trees of *P. chilensis* (0.075%) and soils 20 m away from the trees (0.075%). The amounts of available phosphorus in the soils are higher in soils beneath the trees than in soils 20 m outside the canopies of the trees. They are higher in soils beneath *V. seyal* than in soils beneath the trees of *P. chilensis* and are also 1.5 times higher in soils beneath the trees of *V. seyal* than at 20 m distance from the canopies of the trees. Mean values for iron (Fe^2+^) in soils are 0.25625 20 m from the trunks of trees, 0.44725 at 0 m beneath the trees of *P. chilensis*, and 0.20725 at 0 m beneath *V. seyal*. They are significantly higher in soils beneath *P. chilensis* than in soils at 20 m distance from the trunks and in soils beneath *V. seyal*.

The results of OM measurements are shown in **Figure 1J**. Statistical analysis (Kruskal, pV = 0.19 > 0.05) shows that OM values in soils do not vary significantly according to treatment (species and/or distance). Mean percent values of OM are 1.025 in soils 20 m away from the trunks of trees, 1.5 in soils at 0 m beneath *P. chilensis*, and 2.35 in soils at 0 m beneath *V. seyal*. They are higher in soils beneath the trees than in soils outside the canopies 20 m away from the trees, and they are higher in soils beneath *V. seyal* trees than in soils beneath *P. chilensis* trees. The OM values in soils beneath *V. seyal* are more than twice higher compared with soils 20 m from the trunks of trees.

The results of the C/N ratio measurements are shown in **Figure 1K**. C/N values do not vary significantly (ANOVA, pV = 0.431 > 0.05). The mean values are 11.5 in soils 20 m from the trunks of trees, 9.825 in soils at 0 m beneath *P. chilensis*, and 10.475 in soils at 0 m beneath *V. seyal*.

The results of measurements of total phosphorus (total P_2_O_5_) percentages in soils are shown in **Figure 1L**. Statistical analysis (ANOVA, pV = 0.0699 > 0.05) shows that the percentage of total phosphorus in soils does not vary significantly according to species factor. The mean values of total phosphorus percentages in soils are 0.0175 20 m from the trunks of trees, 0.0425 at 0 m beneath *P. chilensis*, and 0.03 at 0 m beneath *V. seyal*. They are higher in soils beneath the trees than in soils 20 m outside the canopies of the trees. They are higher in soils beneath *P. chilensis* trees than in soils beneath *V. seyal* trees. They are almost 2.5 times higher in soils beneath *P. chilensis* than in soils outside the canopies of the trees.

The results of soil magnesium (Mg^2+^) content measurements are shown in **Figure 1M**. Statistical analysis (ANOVA, pV = 0.334 > 0.05) shows that quantities of Mg^2+^ in soils do not vary significantly according to treatment (species and/or distance to the trees). The average values of quantities of Mg^2+^ in the soils are 1.575 at 20 m distance from the trunks of trees, 2.875 at 0 m beneath *P. chilensis*, and 1.8 at 0 m beneath *V. seyal*. They are higher in soils beneath *P. chilensis* trees and almost equal in soils beneath *V. seyal* trees and outside the canopies 20 m from the trees.

The results of the measurements of calcium (Ca^2+^) content in soils are shown in **Figure 1N**. Statistical analysis (ANOVA, pV = 0.0735 > 0.05) shows that quantities of Ca^2+^ in soils do not vary significantly according to treatment (species and/or distance to trees). The average values of quantities of Ca^2+^ in the soils are 1.1 at 20 m from the trunks of trees, 1.525 at 0 m beneath *P. chilensis*, and 2.3 at 0 m beneath *V. seyal*. They are higher in soils under the trees than in soils 20 m outside the canopies of the trees. They are higher in soils beneath *V. seyal* than in soils beneath *P. chilensis*. They are twice as high in soils beneath *V. seyal* compared with soils at 20 m outside the canopies of the trees.

The results of the measurements of soil manganese (Mn^2+^) contents are shown in **Figure 1O**. Statistical analysis (ANOVA, pV = 0.293 > 0.05) shows that the amounts of Mn^2+^ in soils do not vary significantly according to treatment (species and/or distance to the trees). However, they are higher in soils under the trees than in the soils 20 m from the trunks of trees. They are slightly higher in soils beneath *P. chilensis* than in soils beneath *V. seyal*. The results of the measurements of copper (Cu) and zinc (Zn) in soils are zero and, therefore, have not been plotted.

The results of the CEC measurements in soils are shown in **Figure 1P**. Statistical analysis (ANOVA, pV = 0.12 > 0.05) shows that the cation exchange capacity in soils does not vary significantly according to treatment (species and/or distance to the trees). The average CEC values in soils are 3 at 20 m distance from the trunks of trees, 4.5 at 0 m beneath *P. chilensis*, and 5.8 at 0 m beneath *V. seyal*. They are higher in soils under the trees than in soils 20 m from the trunks of trees. They are higher in soils beneath *V. seyal* than in soils beneath *P. chilensis*. They are almost twice as high in soils beneath *V. seyal* trees compared with soils 20 m from the canopies of the trees.

**Figure 3:**
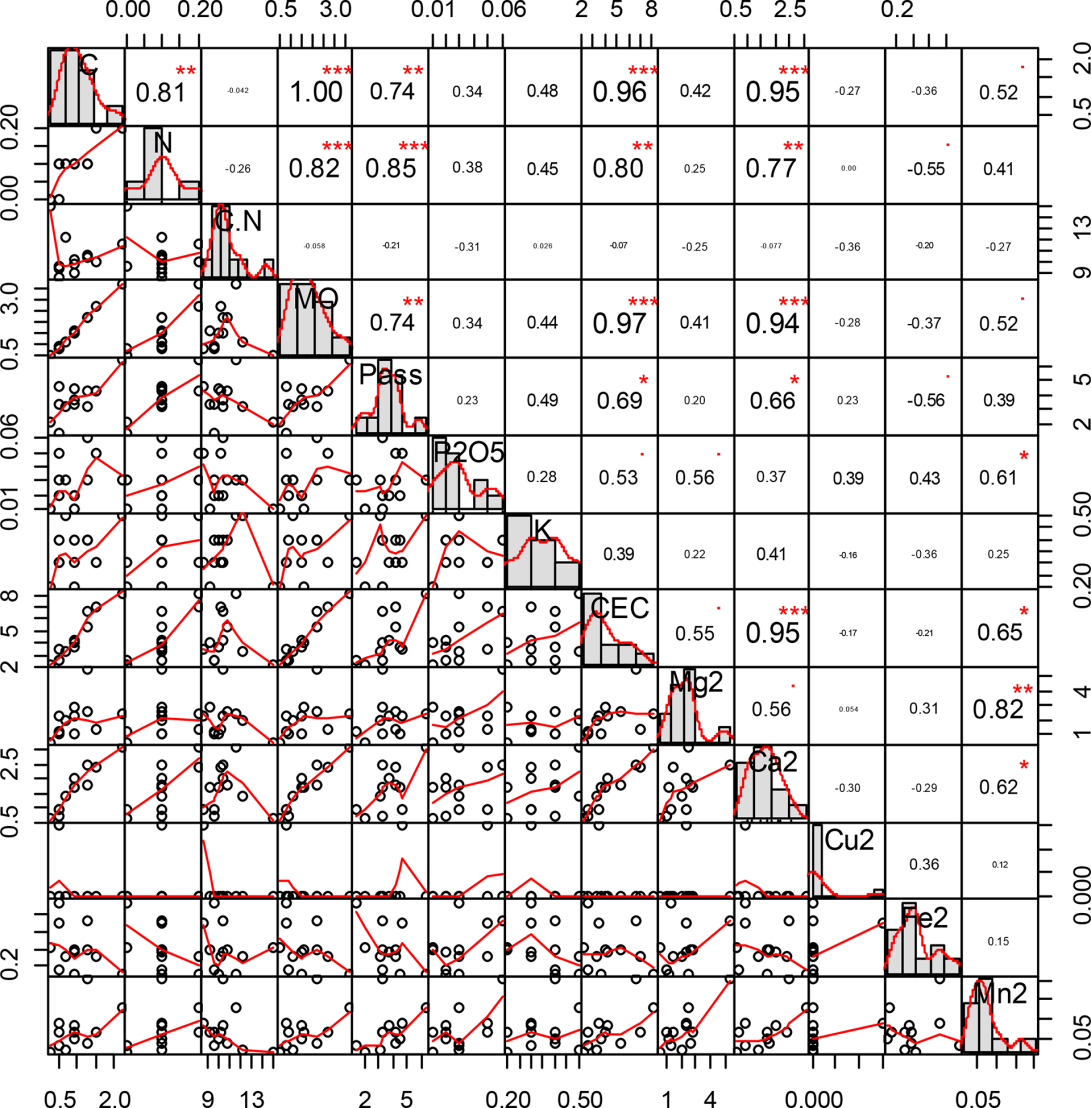
Correlation indexes between carbon, nitrogen, available phosphorus (P ass), and cation exchange capacity (CEC). “*” = Statistically significant difference at 5%, "**" = Statistically significant difference at 1% and "***" = Statistically significant difference 0.1%.

The correlation indexes (**Figure 3**) show a strong link between carbon, nitrogen, available phosphorus (P ass), and CEC. Calcium content also shows links with carbon content, nitrogen content, available phosphorus, OM, and CEC.

**Figure 4:**
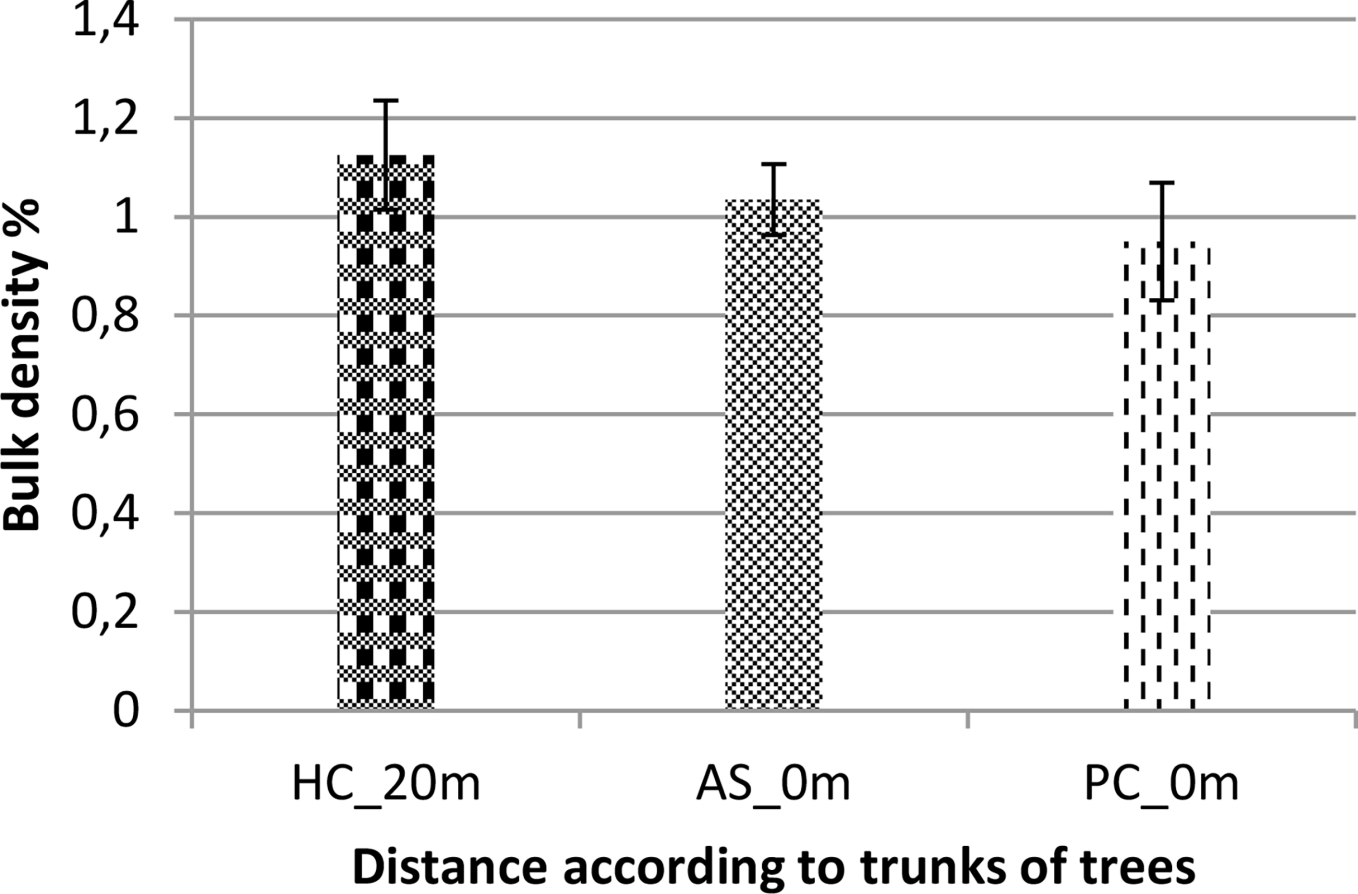
Soil bulk density based on the distance to the trunks of trees. AS, *Vachellia seyal*; Pc, *Prosopis chilensis*; HC, out of cover of trees.

The results of bulk density measurements of soils are shown in **Figure 4**. Statistical analysis (ANOVA, pV = 0.509 > 0.05) shows that the bulk density of soils does not vary significantly according to treatment (species and/or distance to the trees). It was slightly higher in soils 20 m from the canopies than in soils under the trees. For the results of soil moisture measurements, statistical analysis (ANOVA, pV = 0.206 > 0.05) shows that soil moisture values do not vary significantly according to treatment (species and/or distance to the trees). The mean soil moisture values obtained are 0.20475% beneath *V. seyal* trees, 0.052 beneath *P. chilensis*, and 0.054 at 20 m outside the canopies of the trees. They are almost four times higher in soils beneath *V. seyal* than in soils beneath *P. chilensis* and in soils at 20 m from the trees.

### Mycorrhizal inoculum potentials

#### Natural living root AMF colonization

**Figure 5:**
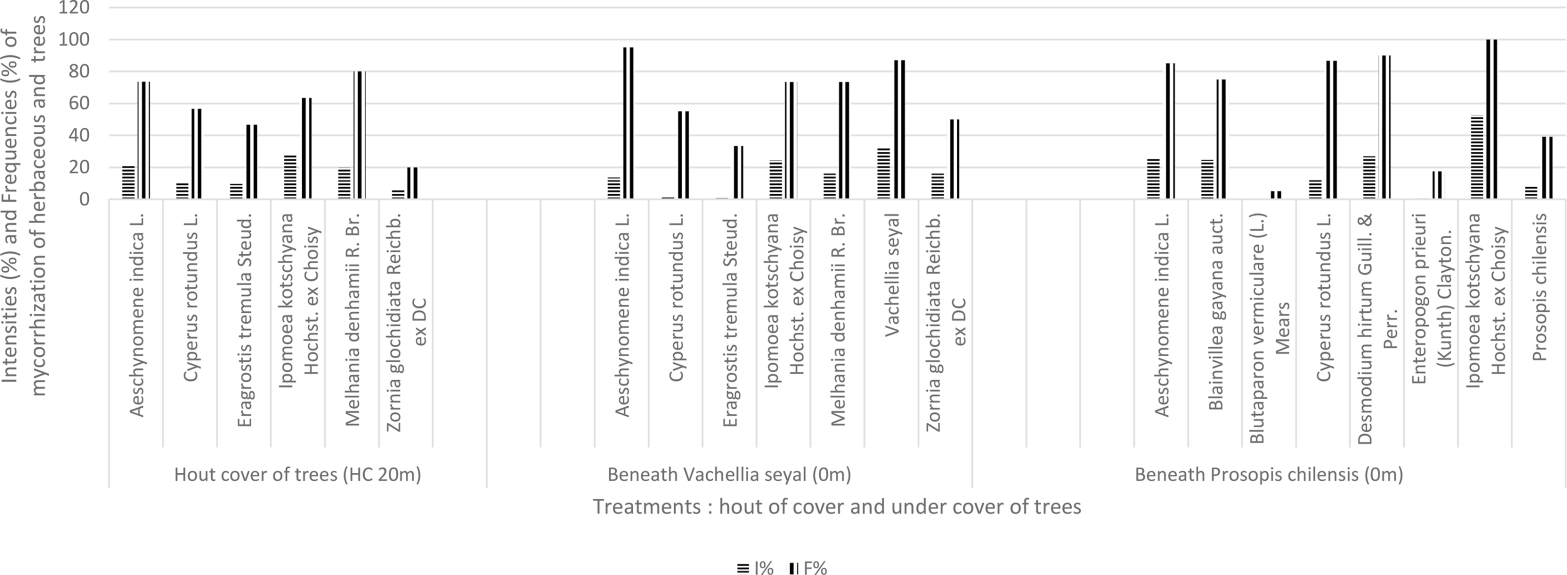
Contribution of herbaceous and tree species to FM and IM (VS, *Vachellia seyal*; PS, *Prosopis chilensis*).

The results of natural living root AMF colonization representing the contribution of herbaceous species in mycorrhization potential in terms of frequencies of mycorrhization (FM) and intensities of mycorrhization (IM) are shown in **Figure 5**. *Vachellia seyal* has higher root FM and IM than *P. chilensis*. The FM and IM of *Z. glochidiata* are higher beneath *V. seyal* than outside the canopies of the trees. *Melhania denhamii* has almost the same mycorrhization rate beneath *V. seyal* than outside the canopies of the trees. *Eragrostis tremula* has a better mycorrhization rate outside the canopies of the trees than beneath *V. seyal*. *Ipomea kotschyana* and *A. indica* have higher FM but with lower IM beneath *V. seyal* than outside the canopies. *Cyperus rotundus* has a slightly better rate outside the canopies than beneath *V. seyal*. *Ipomoea kotschyana*, *A. indica*, and *C. rotundus* have much higher rates beneath *P. chilensis* than beneath *V. seyal* and outside the canopies.

**Figure 6:**
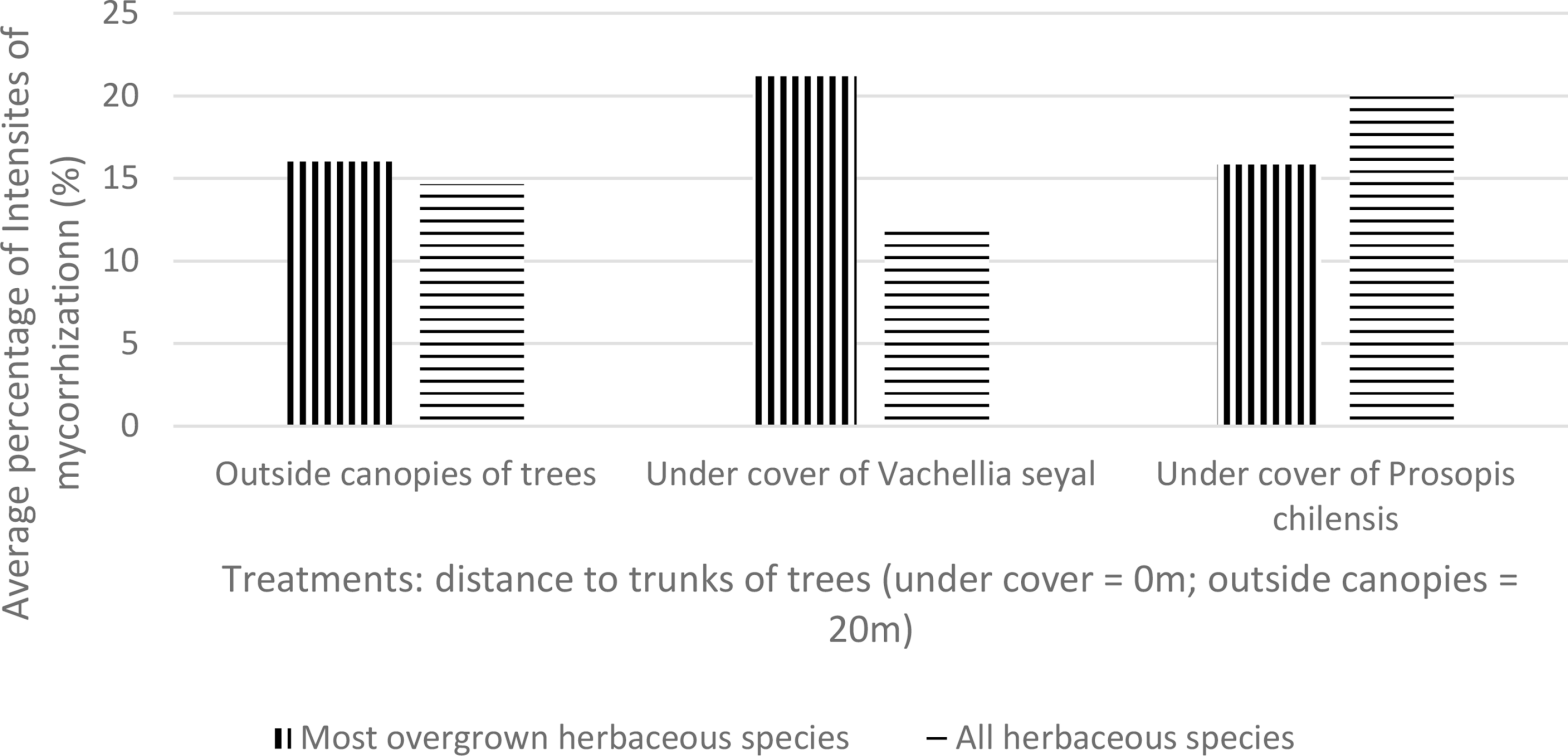
Mycorrhization rate of natural living roots of herbaceous species beneath the trees and outside the canopies of trees.

The average IM values of the living roots of the herbaceous species collected directly from the site outside and under the canopies of the trees are shown in **Figure 6**. The results show that the IM of most overgrown herbaceous species are almost identical under *P. chilensis* and outside the canopies of the trees, but they are higher for the soil beneath *V. seyal*. The IM of the most overgrown herbaceous species are also higher compared with the mean IM of all herbaceous species for the soils beneath *V. seyal*. The mean IM of all herbaceous species are higher than the average IM of most overgrown herbaceous species for soil beneath *P. chilensis*.

**Figure 7:**
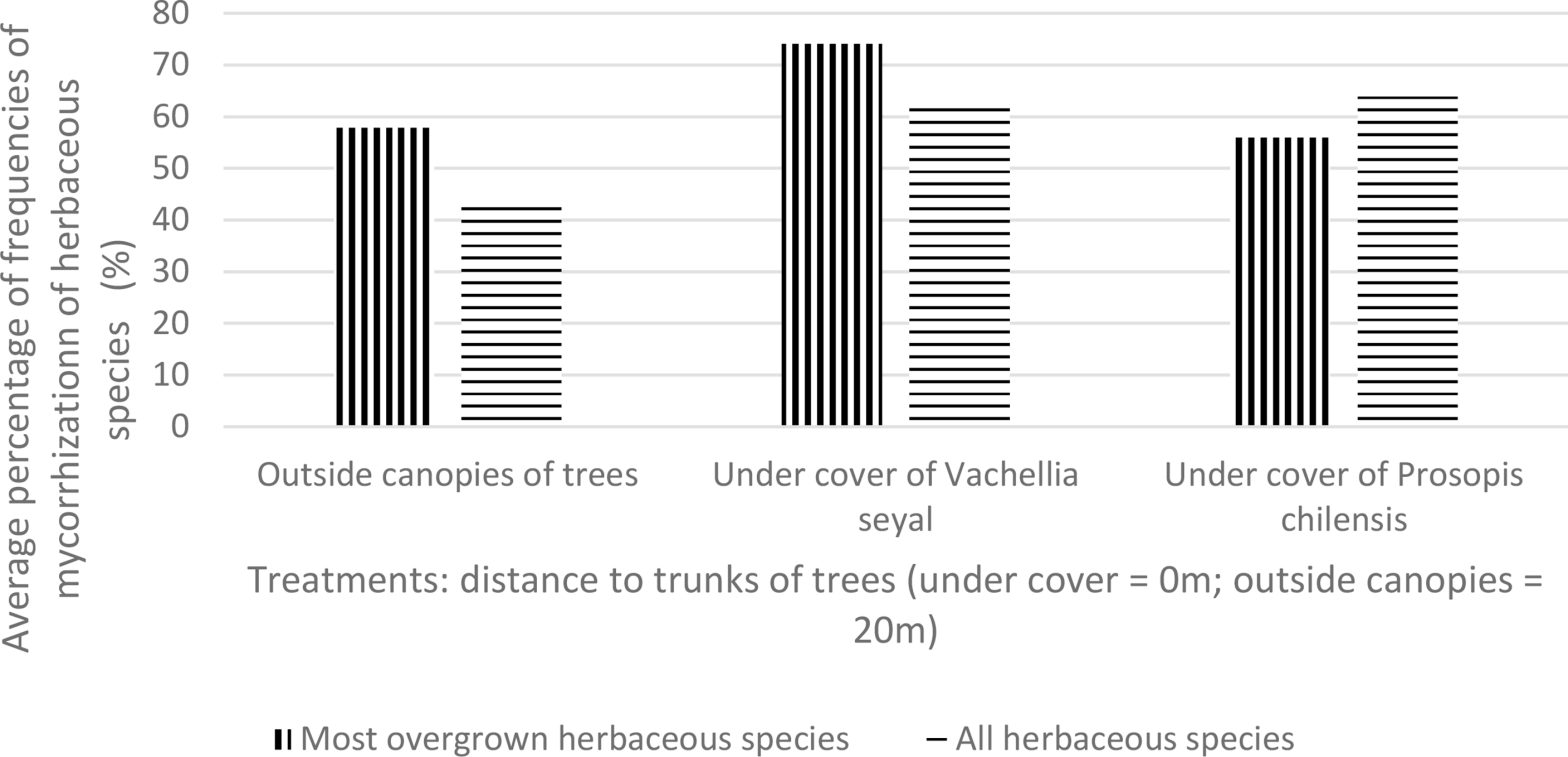
Average mycorrhization rate of natural living roots of herbaceous species with the highest cover indexes beneath the trees and outside the canopies of trees.

**Figure 7** shows the averages of FM for the most overgrown herbaceous species (*Z. glochidiata*, *E. tremula*, and *I. kotschyana* outside the canopies of the trees; *I. kotschyana*, *A. indica*, *C. rotundus*, *M. denhamii, E. tremula*, and *Z. glochidiata* under the canopies of the trees of *V. seyal*; and *D. hirtum*, *I. kotschyana*, *B. vermiculare*, *E. prieuri*, *B. gayana*, *A. indica*, and *C. rotundus* under the canopies of the trees of *P. chilensis*). These results show that the average values of most overgrown herbaceous species are higher beneath *V*. *seyal* and decrease outside the canopies of trees and beneath *P. chilensis*. On the other hand, the mean values of FM of all herbaceous species are almost equal beneath *P. chilensis* and beneath *V. seyal*.

**Figure 8:**
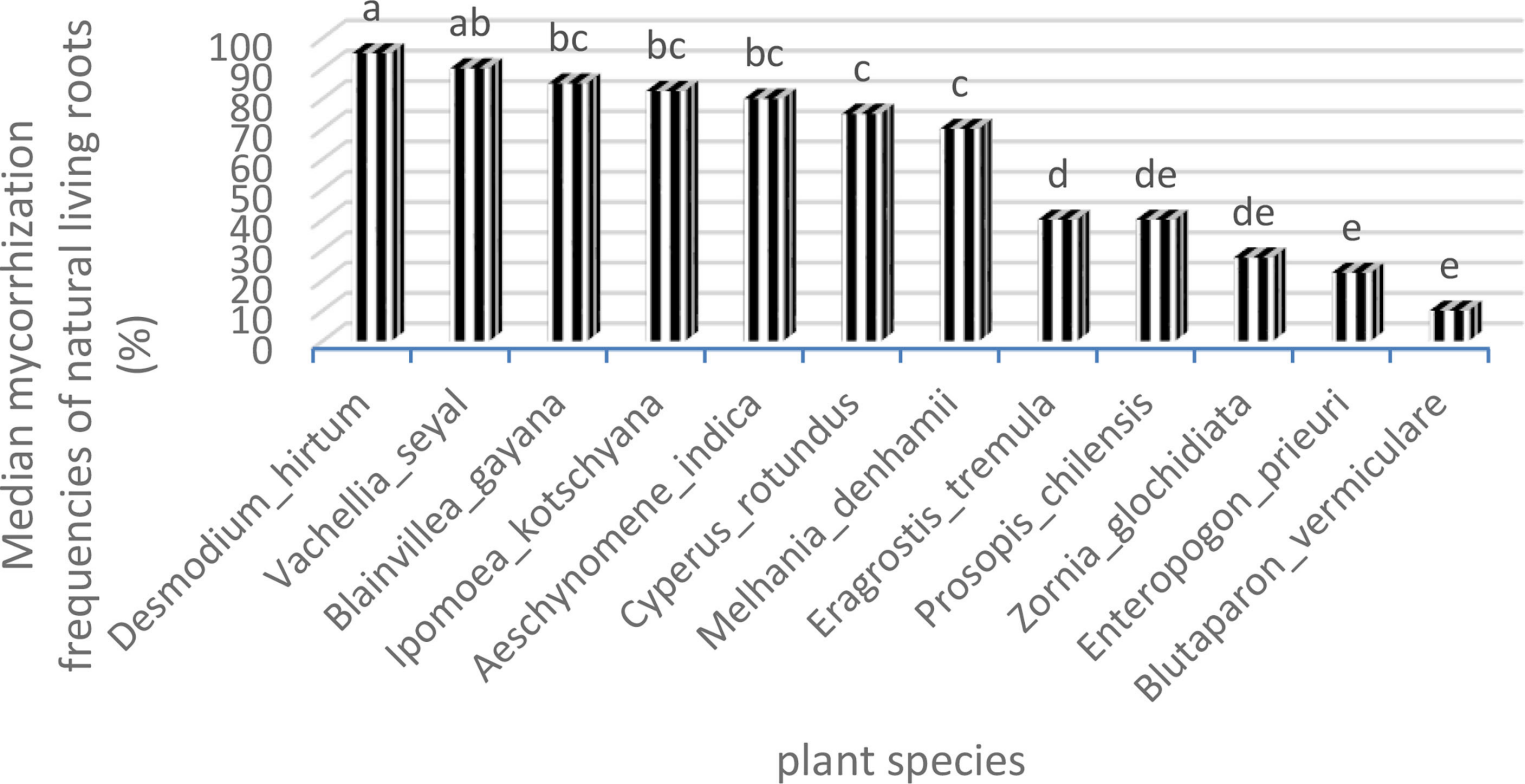
Frequencies of mycorrhization of natural living roots. Means with identical letters are statistically equivalent at the 5% level.

**Figure 9:**
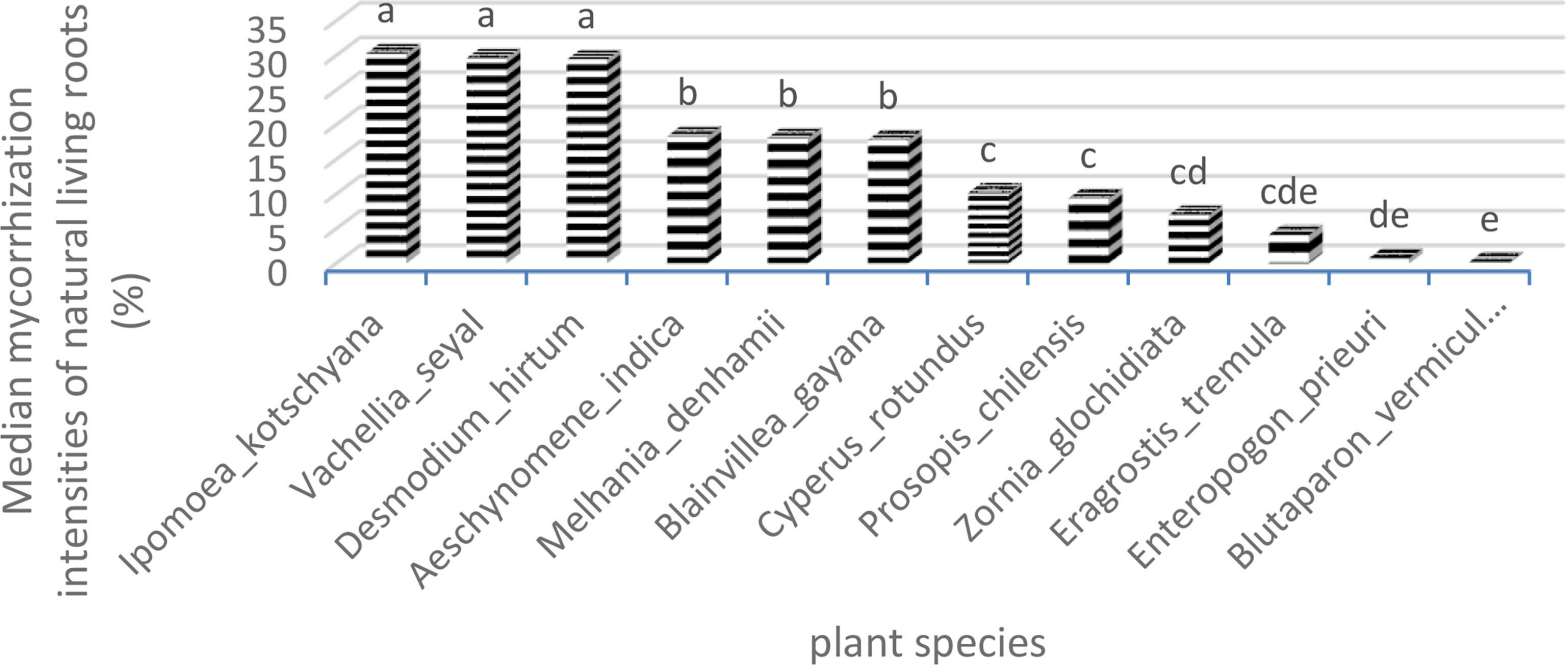
Intensities of mycorrhization of natural living roots. Means with identical letters are statistically equivalent at the 5% level.

**Figures 8** and **9** show also the natural AMF mycorrhization of grasses and trees. The non-parametric median comparison tests applied to FM and IM allowed the identification of groups for both cases. The FM allowed the identification of two groups (**Table 9**). The first group comprises *D. hirtum*, *V. seyal*, *B. gayana*, *I*. *kotschyana*, *A. indica*, *C. rotundus*, and *M. denhamii* and the second group consists of *E. tremula*, *P*. *chilensis*, *Z. glochidiata*, *E. prieuri*, and *B. vermiculare*. The FM of the first group are higher or equal to 70% and those of the second group are lower or equal to 40%. *Vachellia seyal* belongs to the first group and *P. chilensis* belongs to the second group.

**Table 9:** Tree–herbaceous plant groupings according to mycorrhization frequencies of natural living roots.

Species	FM	Groups
*Desmodium hirtum*	95	a
*Vachellia seyal*	90	ab
*Blainvillea gayana*	85	bc
*Ipomoea kotschyana*	82.5	bc
*Aeschynomene indica*	80	bc
*Cyperus rotundus*	75	c
*Melhania denhamii*	70	c
*Eragrostis tremula*	40	d
*Prosopis chilensis*	40	de
*Zornia glochidiata*	27.5	de
*Enteropogon prieuri*	22.5	e
*Blutaparon vermiculare*	10	e

FM, frequencies of mycorrhization. Means with identical letters are statistically equivalent at the 5% level.

The comparisons of medians applied to the IM allowed the identification of three groups (**Table 10**). The first group comprises *I. kotschyana*, *V. seyal*, and *D. hirtum* with intensity values between 29.4% and 30.7%. The second group comprises *A. indica*, *M. denhamii*, and *B. gayana* with intensity values between 17.8% and 18.36%. The third group comprises *C. rotundus*, *P. chilensis*, *Z. glochidiata*, *E. tremula*, *E. prieuri*, and *B. vermiculare* with intensity values between 0.2% and 10.17%. The first two groups are more homogeneous in terms of IM values, and the third group is more heterogeneous with lower IM values. The groups defined according to the FM are more heterogeneous than those defined according to the IM.

**Table 10:** Tree–herb grouping as a function of intensities of mycorrhization of living root.

Species	IM	Groups
*Ipomoea kotschyana*	30.27	a
*Vachellia seyal*	29.6	a
*Desmodium hirtum*	29.4	a
*Aeschynomene indica*	18.36	b
*Melhania denhamii*	18.11	b
*Blainvillea gayana*	17.8	b
*Cyperus rotundus*	10.17	c
*Prosopis chilensis*	9.35	c
*Zornia glochidiata*	6.95	cd
*Eragrostis tremula*	4.105	cde
*Enteropogon prieuri*	0.75	de
*Blutaparon vermiculare*	0.2	e

IM, intensities of mycorrhization. Means with identical letters are statistically equivalent at the 5% level.

#### Most probable number of AM fungi community

**Figure 10:**
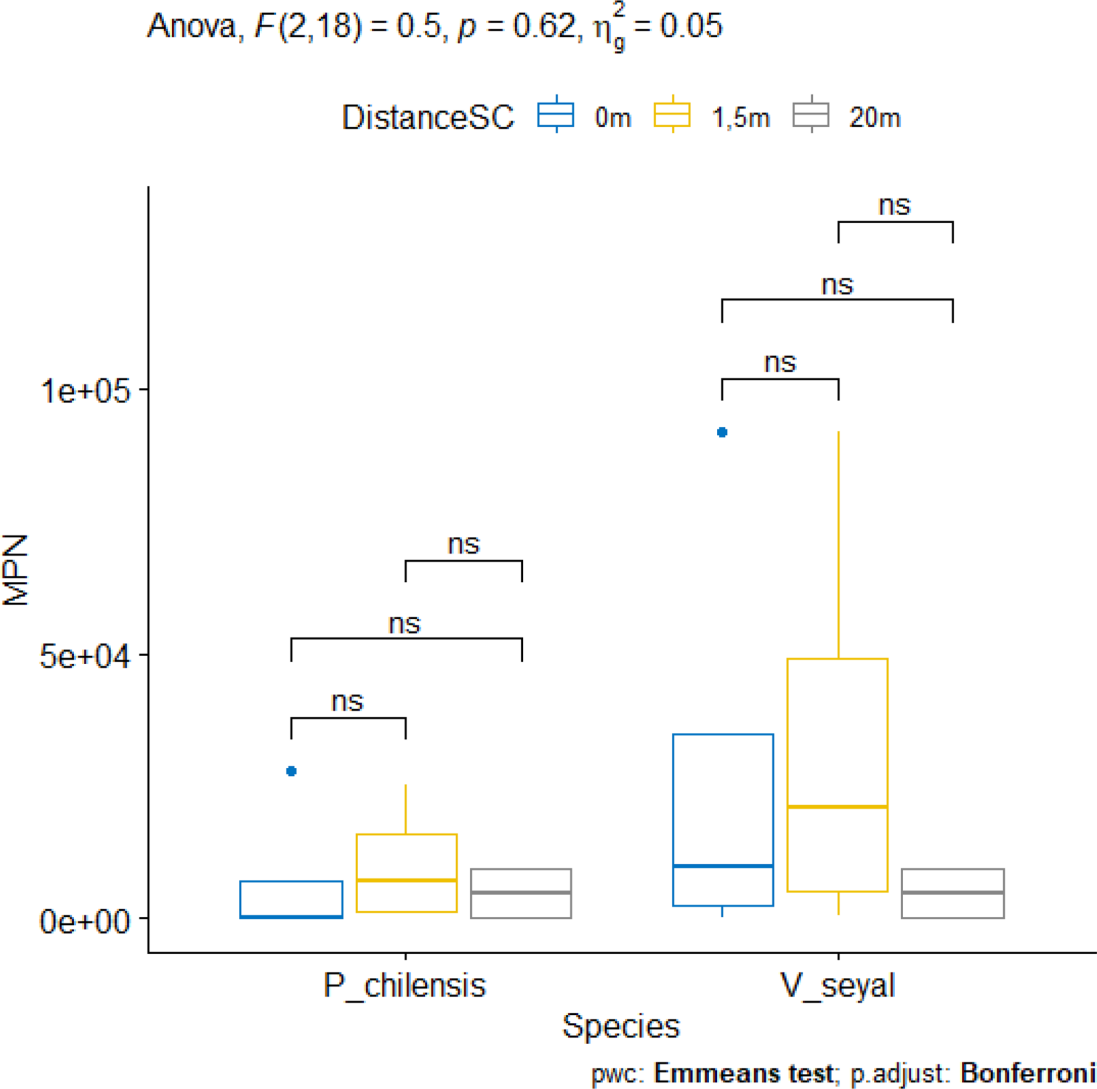
Most probable number (MPN) of propagules in soil beneath the trees and according to the distance from the trunk. *ns* = non-significant.

The results of the MPN of AMF propagules in soil capable to generate mycorrhizae are represented in **Figure 10**. The MPN for soils beneath the trees at distances of 0, 1.5, and 20 m from the trunks of trees did not show significant differences for each species. However, values are higher in soils beneath *V.* seyal than in soils beneath *P. chilensis*. For both *V. seyal* and *P. chilensis* species, MPN values are higher in soils 1.5 m from tree trunks.

#### Symbiotic root colonization by AM fungi community in soil collected beneath the trees and outside of the canopies

**Figure 11:**
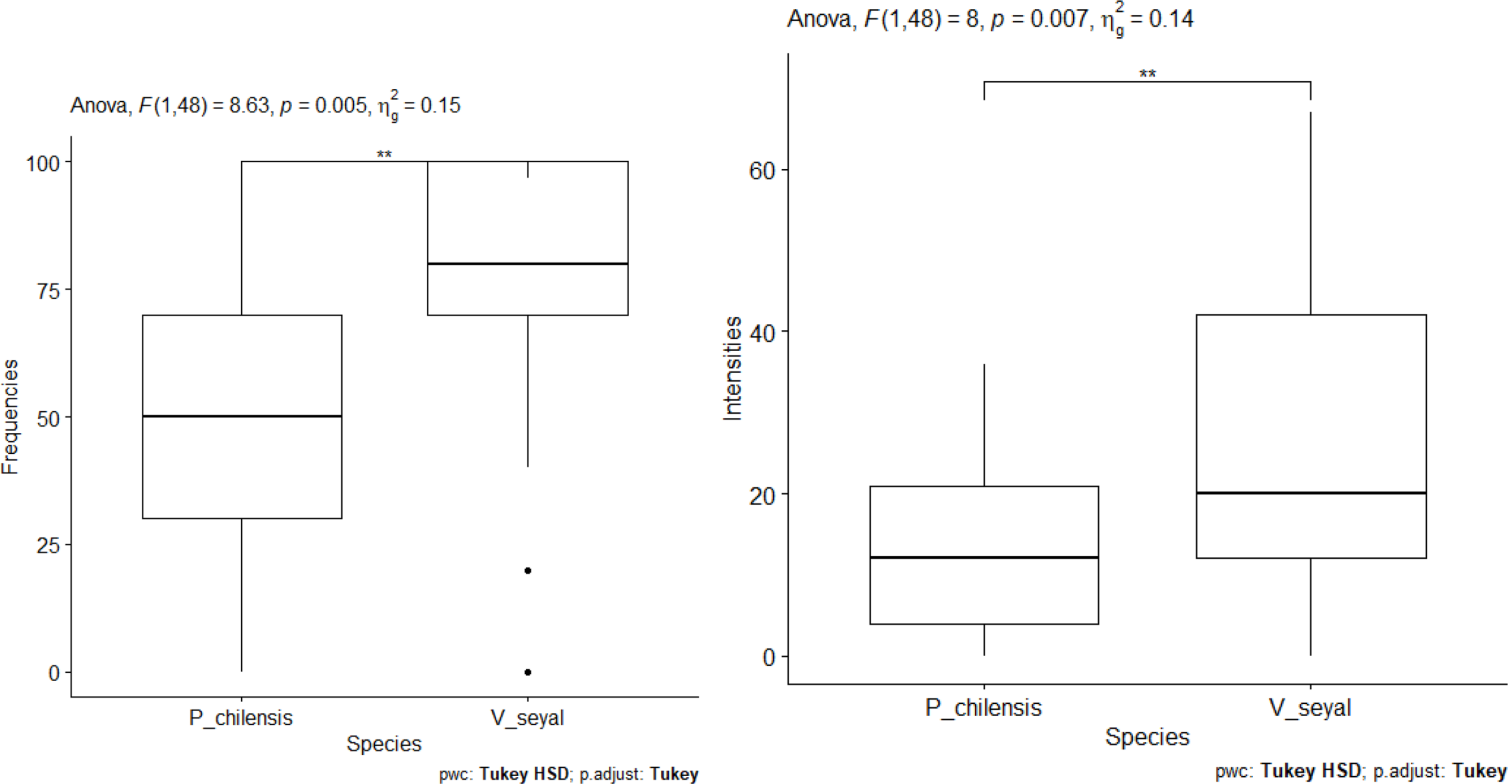
Frequencies of mycorrhization and intensities of mycorrhization according to tree species. "**" = Statistically significant difference at 1%.

**Figure 12:**
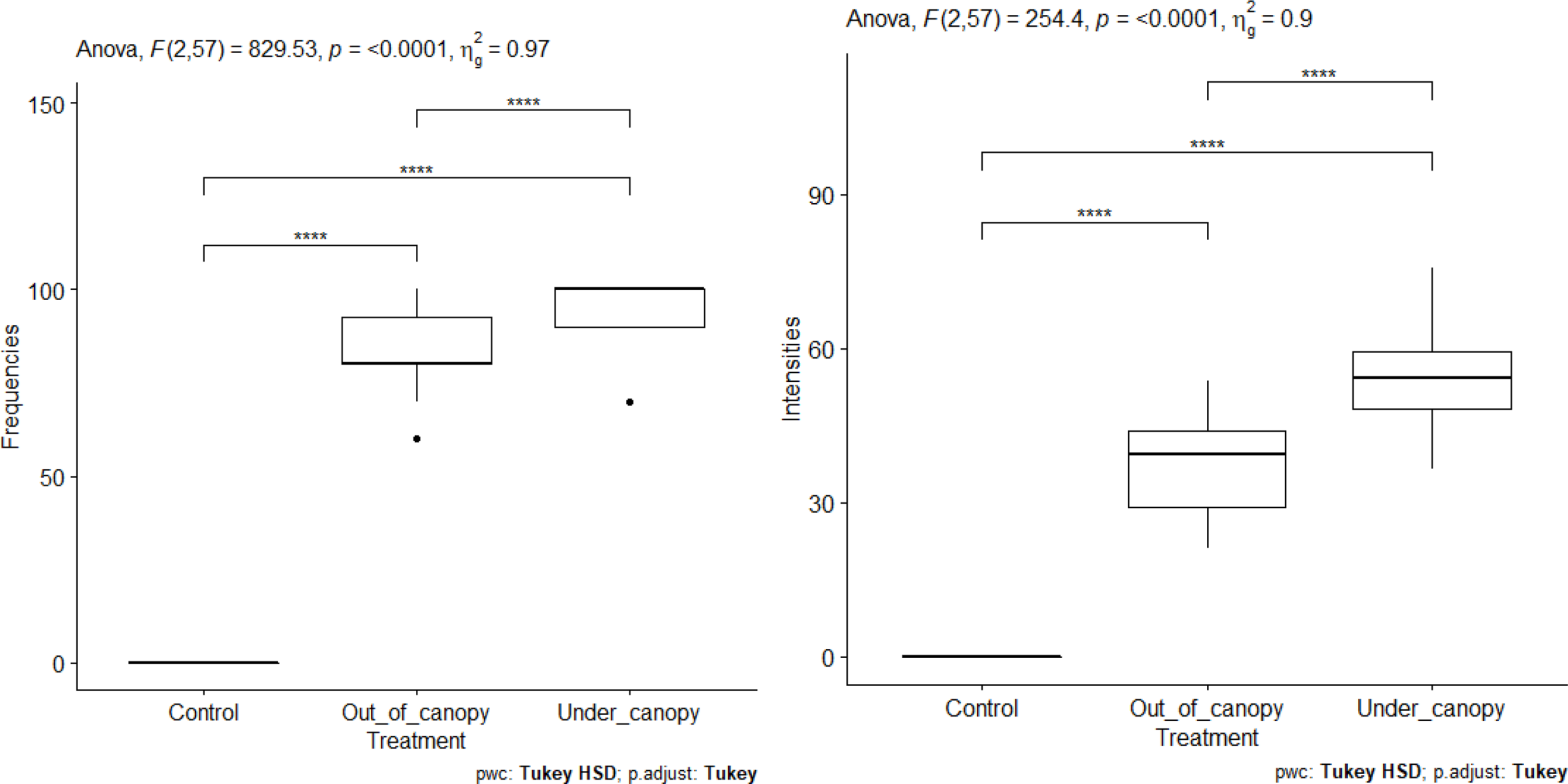
Frequencies of mycorrhization and intensities of mycorrhization according to treatment out of the tree canopy and under the tree canopy. "****" = Statistically significant difference 0.1%.

The results of the measurement of FM and IM according to tree species are shown in **Figure 11**. The FM and the IM show significant differences between species (**Figure 11**). They are higher in the roots of plants grown on soil from beneath *V. seyal* than in the roots of plants grown on soil from beneath *P. chilensis*. The estimated FM are about 50% beneath *P. chilensis* but over 75% beneath *V. seyal*. The IM values are slightly above 10% for the roots of plants grown on soil from beneath *P. chilensis* and about 20% for the roots of plants grown on soil from beneath *V. seyal*. Compared with treatments under the trees and outside the canopies of trees (**Figure 12**), the FM and the IM also show significant differences. They are very significantly higher beneath the canopies of trees than outside the canopies.

#### AM root colonization rate in the presence of organic matter

**Figure 13:**
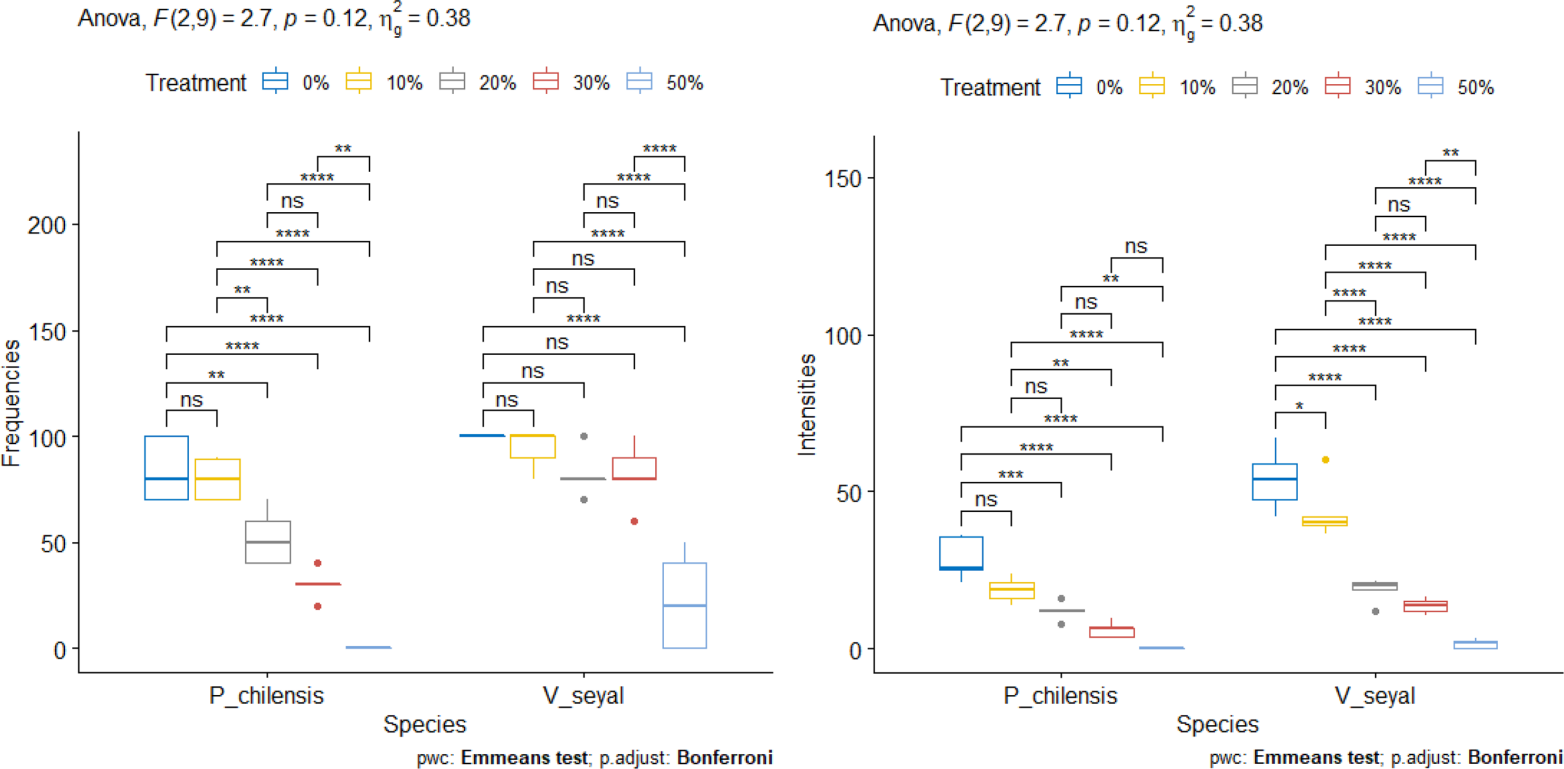
Frequencies of mycorrhization (FM) and intensities of mycorrhization (IM) according to the rate of organic matter. “*” = Statistically significant difference at 5%, “**” = Statistically significant difference at 1% and “***” = Statistically significant difference 0.1% and *ns* = non-significant.

The FM (**Figure 13**) of roots decrease with increasing organic matter in soils beneath *V. seyal* and in soils beneath *P. chilensis*. The FM are higher in the roots of *V. seyal* plants than in the roots of *P. chilensis* plants in all percentages of OM. The FM reached 100% on the roots of *V*. *seyal* plants at 0% OM and then decreased to 94%, 82%, 82%, and 22%, respectively, in the presence of 10%, 20%, 30%, and 50% OM, respectively. For the roots of *P. chilensis* plants, the FM values for the same percentages of OM are 84%, 79.8%, 52%, and 30%, respectively. The increase of soil OM content from 0% to 10% does not have any effect on root FM which is higher in *V. seyal* roots. The increase of soil OM content from 10% to 20% does not affect the FM of *V. seyal* roots, but it decreases significantly the FM of *P. chilensis* roots.

For *V. seyal* plants, significant decreases are noted in root FM between soil OM rate 0% and 50% OM, as well as between 10% and 50%, between 20% and 50%, and between 30% and 50%. The decrease in root FM is not significant with increases in OM ranging from 0% to 30%.

For *P. chilensis* plants, increases in soil OM rate from 0% to 10% do not generate a significant decrease in root FM. However, the increase of soil OM content from 10% to 20% induced a significant decrease in root FM. So, no significant decrease in root FM noted with OM levels from 20% to 30% also means that it will induce a decrease in root FM. However, significant decreases in root FM are noted if there is an increase in OM rate from 0%, 10%, 20%, or 30% to 50% OM. This means that it is not recommendable to fertilize the soil with OM of *P. chilensis* at a rate of 50%. Increases of OM rate up to 30% result in significant decreases in root FM compared to 0% and 10% OM.

The IM values (**Figure 13**) of the roots of both *V. seyal* and *P. chilensis* decrease with the increase of soil OM rate. The IM values are higher in the roots of *V. seyal* plants than in the roots of *P. chilensis* plants in all percentages of OM.

For *V. seyal* plants, increases in OM rate in soil up to 50% decrease significantly the IM. Decreases in the IM are also observed after increases in OM rate at all levels except for the one between 20% and 30% OM.

For *P. chilensis* plants, increases in OM from 0% to 50%, from 10% to 50%, and from 20% to 50% resulted in significant decreases in the IM. The increases in OM rate from 0% to 10% or from 10% to 20% or from 30% to 50% do not result in significant decreases in the IM.

## Discussion

The results of water pH measurements show that soils at 0 m under the trees are slightly more acidic than the soils 20 m from the trunks of trees. The pH values are approximately equal beneath *V. seyal* trees to beneath *P. chilensis* trees. Soil pH values 20 m from the trunks of trees are higher. This difference shown in soil pH could be related to a higher OM content in soil under the trees and more beneath *V. seyal*. OM provides litter and tends to lower soil pH, but it also plays an important role in protecting microorganisms from antimicrobial agents. According to the work of Liu et al. (2018), soil OM is strongly related to vegetation cover, and there are links between vegetation properties and soil characteristics. Similarly, the results of this work show a direct link between the presence of trees and OM resulting in higher OM levels in soils under the trees than at a distance of 20 m from the trunks of trees. OM also constitutes a reserve of carbon and nitrogen in terrestrial ecosystems (Schlesinger, 1997), which would explain higher soil carbon and nitrogen content beneath *V. seyal* trees. Regarding KCl pH, which expresses an acidity reserve, meaning pH values that a soil could take on in the event of a change in conditions (addition of OM, etc.), the values measured show significant differences for soils under the trees and soils 20 m from the trunks of trees. They are lower under the trees than at 20 m distance from the trunks of trees, which reflects smaller variations in pH in the event of a change in the environment but also a greater potential for solubilization of substances. The presence of trees thus has an impact on the alkaline or acidic status of the soil.

Electrical conductivity measurements showed significantly higher values (Kruskal, pV = 0.049< 0.05) for soils 20 m from the trunks of trees and soils beneath *P. chilensis* trees compared with soils collected beneath *V. seyal* trees. These results indicate higher salinity levels in soils 20 m from the trunks of trees and in soils beneath *P. chilensis* trees compared with soils beneath *V. seyal* trees. Referring to the work of Faye et al. (2019), soil 1 outside the tree canopies and soil 1 at 0 m beneath *P. chilensis* can be considered hypersaline as they have respective EC values of 3,200 and 2,870 S cm^−1^ exceeding the threshold of 2,000 S cm^−1^; soil 2 outside the canopies of trees and soil 2 at 0 m beneath *P. chilensis* are very salty as they have respective EC values of 1,690 and 1,799 S cm^−1^ which are within the limits between 1,000 and 2,000 S cm^−1^; soils 1 and 2 at 0 m beneath *V. seyal* are salty to slightly salty with EC values of 817 and 276 S cm^−1^. The presence of *V. seyal* trees would have a significant effect in reducing the salinity level in soils. The lower salinity levels in soils beneath *V. seyal* trees compared with soils beneath *P. chilensis* trees and uncovered soils may also explain the presence of more herbaceous plants with higher density beneath *V. seyal* trees than beneath *P. chilensis* trees and uncovered soils. The presence of *V. seyal* lowers the salinity level and, thus, increases the species richness of the grasses. It would then have the potential to restore the vegetation cover because soils with the same numbers have the same distance to the tans and should have similar salinity levels. If this is not the case, it means that the presence of the trees has had an effect on salinity. The EC levels recorded in the soils at 0 m beneath *V. seyal* correspond to values at which the herbaceous layer, the shrub layer, and the tree layer of the flora can develop. The higher salinity levels beneath *P. chilensis* compared with outside the canopies of trees indicate that *P. chilensis* concentrates salt, which may contribute to a reduction in herbaceous density beneath *P. chilensis*.

Soil sodium (Na^+^) contents (Kruskal, pV = 0.041< 0.05) varied significantly if comparing soils beneath *V. seyal*, soils beneath *P. chilensis*, and soils 20 m from the trunks of trees. Sodium levels are higher in soils collected beneath *P. chilensis* trees and in soils collected at a distance of 20 m from the trunks of trees. The average Na^+^ soil content is 1.875 meq/100 g at 20 m from the trunks of trees, 2 meq/100 g at 0 m beneath *P. chilensis*, and 0.825 meq/100 g at 0 m beneath *V. seyal* trees. Sodium contents are more than two times lower in soils beneath *V. seyal* trees compared with soils beneath *P. chilensis* trees and in soils 20 m outside the canopies of trees. These results on sodium levels confirm those on electrical conductivity showing lower salinity levels in soils beneath *V. seyal* trees. Furthermore, Pearson’s correlation index of 0.84 indicates that soil sodium content is strongly related to electrical conductivity. The presence of *V. seyal* trees would thus reduce soil Na^+^ content by a factor of more than 2. On the other hand, the presence of *P. chilensis* trees seems to increase soil sodium content. This fact also explains the greater number of herbaceous plants with a greater plant cover present beneath *V. seyal* trees than beneath *P*. *chilensis* trees.

Available phosphorus levels vary significantly and are higher in soils under the trees than in soils 20 m outside the canopies of trees. These higher levels of available phosphorus for soils beneath *V. seyal* trees reflect the effects of trees on phosphorus availability. Indeed, the presence of trees also generates the presence of herbaceous plants that associate with AMF, which solubilize phosphorus and make it available to plants. These symbiotic associations are confirmed by the results on higher mycorrhizal potentials in soils under the trees. Microorganisms decompose plant debris, thus contributing to increasing soil phosphorus levels. Zhang et al. (2016) report that increasing vegetation cover is beneficial for increasing phosphorus. Liu et al. (2018) also showed a positive correlation between vegetation cover and total soil phosphorus content. These observations are in line with the results of this study which shows higher levels of total phosphorus under the trees than at 20 m from the trunks of trees. Total phosphorus contents are higher in soils beneath *P. chilensis* than in soils beneath *V. seyal*, which could mean that the presence of *V. seyal* has an effect on phosphorus uptake which will result in lower total phosphorus levels under its trees. This effect would be by a factor of 1.39. When comparing total phosphorus levels under the trees, it is 1.41 times higher beneath *P. chilensis* than beneath *V. seyal*. Total phosphorus levels beneath *P. chilensis* are almost 2.5 times higher than under the trees. The analysis of results also shows that the presence of trees contributes to increased phosphorus levels in soils; this could be a result of the decomposition of plant litter. In saline soils, phosphate ions generally precipitate with Ca^2+^, Mg^2+^, and Zn^2+^ ions and, thus, become less available to plants (Azcón-Aguilar et al., 1979). Thus, in addition to the contribution of AMF in making phosphorus available, salinity levels become lower under the trees (lower electrical conductivity) and, particularly, beneath *V. seyal* would be more favorable for phosphorus availability in soils under the trees compared with soils 20 m outside the canopies of trees with higher EC. The acidic status of the soils is also favorable to phosphorus solubilization, and this is reflected in this study by higher levels of available phosphorus in soils under the trees, which also have lower pH. These conditions could prevent the precipitation of important ions (including phosphorus) caused by salinity (Azcón-Aguilar et al., 1979) and make them available to plants, which is essential to help alleviate stress (Cantrell and Linderman, 2001).

Analysis of the results shows that the carbon content is significantly higher in soils under the trees than at distances of 20 m from the trunks of trees. Soils beneath *V. seyal*, receiving OM input from deciduous leaves, contain up to 1.5 times more carbon and more than twice as much nitrogen as soils beneath *P. chilensis*, whose leaves are more rarely deciduous. They also contain up to more than twice as much carbon and nitrogen as soils 20 m from the trunks of trees. Exudates from the roots of *P. chilensis* also contribute to soil carbon content; this fact may explain the much more carbon in soils beneath *P. chilensis* than in soils 20 m from the trunks of trees which do not benefit as much from the presence of trees.

The C/N ratio is a good indicator of inorganic nitrogen retention (Templer et al., 2012). This is because OM deposited in soil is degraded by microorganisms. In this study, the C/N ratios below 25 mean that microorganisms release nitrogen during the decomposition of OM in the soil. The higher C/N values beneath *V. seyal* and out of cover correlate positively with the fact that the corresponding soils have a sandier texture and are consistent with the results of the studies of Marty et al. (2017) and Callesen et al. (2007). The C/N values of about 10 under the canopies of trees reflect microbial OM decomposition activity with an increasing rate.

Soil iron contents shows significant differences. Iron levels are higher in soils beneath *P. chilensis* than in soils beneath *V. seyal* and in soils 20 m outside the canopies of trees. They are lower in soils beneath *V. seyal*.

The CEC of soils shows a high correlation coefficient with the levels of carbon, nitrogen, OM, and available phosphorus with values of 0.96, 0.80, 0.97, and 0.69, respectively. The CEC of soils did not show significant differences. However, the CEC is higher under the trees than at a distance of 20 m from the trunks of trees; this reflects a higher soil fertility potential under the trees than 20 m from the trunks of trees. It is higher beneath *V. seyal* trees than beneath *P. chilensis* trees and is favorable for the availability of K^+^, Ca^2+^, and Mg^2+^ ions to plants in these soils because their recharge into the soil solution is better.

Regarding bulk density and moisture, statistical analyses (ANOVA, pV = 0.509 > 0.05) do not show significant differences between soils. However, the presence of AMF under the trees favors the formation of aggregates in the soil (Wright and Upadhyaya, 1998). AMF mycelia also play an important role in the formation of stable aggregates in the soil (Miller and Jastrow, 2000). In contrast, moisture is almost four times higher beneath *V. seyal* trees than beneath *P. chilensis* and 20 m from the trunks of trees; this is more favorable for maintaining the abundance and diversity of soil microflora.

The site where floristic surveys were carried out shows a floristic and ecological homogeneity. These two requirements (repetition of *V. seyal* and *P. chilensis* individuals and existence of saline stress in the area) mean that the analysis of the data should provide helpful information for the management of this ecosystem. In the previous surveys conducted, dicotyledons were more represented than monocotyledons under the trees and outside the canopies of trees. The survey similarity coefficients of 0.57 calculated between surveys beneath *V. seyal* and surveys beneath *P. chilensis* and 0.52 between each of these two above types of survey and surveys outside the canopies of trees show that they have many species in common by referring to the results of the study of de Bello et al. (2007), and therefore, a significant number of species occur in the areas where the surveys were conducted. This reflects biodiversity and that environmental conditions under the canopies of trees and outside the canopies would be similar. Species recorded could then be quite authentic to this site and would generally tolerate the main ecological factors. The main differences are in their abundance and dominance in specific locations that have particular conditions that favor the development of one or another species. Exploitation of their potential could be implemented in programs for the recovery of land affected by salinization. Under the canopies of *V. seyal* trees, the most frequently found herbaceous species have the same pairwise association coefficients and would be related to specific conditions under the trees. In contrast, under the canopies of *P. chilensis*, the association indexes between herbaceous species are variable, thus showing different preferences toward the presence of trees. The analysis of presence–absence results shows that a total of 29 species were recorded. The specific richness of species is variable depending on whether the surveys were taken under or outside the canopies of trees. The results showed that the presence of trees has a favorable effect on herbaceous vegetation, compared with control outside the canopies of trees, because it improves the species diversity. The number of species per survey is higher under the trees than outside the canopies of trees. It is higher beneath *V. seyal* than beneath *P. chilensis*. The presence of trees thus seems to have a positive influence on the number of herbaceous species present. The presence of trees provides litter that improves soil quality and availability of nutrients, favoring soil fertilization and, therefore, favorable development of grasses under the trees (Remigi et al., 2008; Diallo et al., 2013). Higher soil moisture under the trees favors the development of grasses. If we compare the structure (floristic or species richness) under the canopies of *V. seyal*, there are 24 genders and 25 species; under the canopies of *P. chilensis*, there are 19 genders and 19 species, whereas outside the canopies of trees, there are 15 genders and 16 species. These results reflect a higher diversity beneath *V. seyal* than beneath *P. chilensis*, and the lowest diversity is found outside the canopies of trees. The similarity analysis also revealed seven groups among the herbs. This abundance of herbaceous plants under the canopies of *V. seyal* could also be explained by the microbial potential in the rhizosphere of trees but also by a sharing of mutualistic networks such as that of AMF. These beneficial effects would favor the establishment of several species, some of which are more abundant because they grow more rapidly and have more efficient occupancy strategies.

In terms of sociability, excluding *E. tremula* and *E. prieuri*, herbaceous plants perform better beneath *V. seyal* trees than outside the canopies of the trees and beneath *P. chilensis*. *Melhania denhamii* and *D. hirtum* seem to be more associated with *V. seyal*. They would be elective species favored there by a selective advantage existing in the perimeter around the trunk in a radius of less than 1 m where their individuals are very abundant with high cover rates and more numerous compared with individuals of other species. This abundance under the canopies of trees is very pronounced at the perimeter within a radius of about 2 m from the trunk of trees. This pattern, which is repeated beneath all *V. seyal* trees in the same area of the site, may be related to a preference of these species for association with *V. seyal* trees. This preference could be related to a specificity of this part beneath the trees because beyond this, *M. denhamii* and *D. hirtum* are represented by few individuals at the periphery of canopies and by rare individuals outside the canopies of trees. This confirms the link between *V. seyal* and the presence of *M. denhamii* and *D. hirtum*. This decrease in density of *M. denhamii* and *D. hirtum* individuals is concomitant with the appearance and increase in density of *E. tremula* individuals at the periphery of canopies of *V. seyal* trees. *Eragrostis tremula* exhibits the highest abundance–dominance index outside the canopies of trees, whereas its individuals were not found within a radius of about 1 m around the trunks of *V. seyal* trees. The presence of trees would have an impact on the density of its individuals, which are more numerous outside the canopies than under the trees. *Cyperus rotundus* presents the highest cover under the canopies of *P. chilensis*. It is more closely related to *P. chilensis*, where it alone has the highest AD code and its individuals are scattered under the canopies of *V. seyal* and have a very low cover outside the canopies of trees. This result confirms a preference of *C. rotundus* for association with *P*. *chilensis*. *Enteropogon prieuri* does not seem to be strongly related to *V. seyal* and *P. chilensis* as it has one of the highest cover coefficients outside the canopies with a high AD code, but its individuals are scattered under the canopies of *P. chilensis* and very rare under the canopies of *V. seyal*. In summary, the AD codes of *M. denhamii* and *D. hirtum* decrease from the trunks of *V. seyal* to outside canopies, that of *C. rotundus* decreases from the canopies of *P. chilensis* to outside canopies, and those of *E. tremula* and *E. prieuri* decrease from outside canopy areas to canopy areas.

The association preferences between *V. seyal*, *M. denhamii*, and *D. hirtum* could be due to an AMF shared colonization network conferring an advantage. Indeed, the roots of *V. seyal*, *M. denhamii*, and *D*. *hirtum* showed quite high IM and FM around the trunk of trees, which could justify their association. *Eragrostis tremula*, which has a low IM and a relatively low FM, would not have benefited much from the selective advantage of the AMF shared network between *V. seyal*, *M. denhamii*, and *D. hirtum*. This mycorrhization advantage is less beneath *P. chilensis*, which has low values of IM and FM, as well as *C. rotundus*, which is dominant under its canopy. The other species present (*I. kotschyana* Hochst. ex Choisy, *D. hirtum* Guill. & Perr., *A. indica* L., *B. gayana* auct.), even if they have high IM and FM, have small AD codes and, therefore, would not benefit from their symbioses. In addition, the low herbaceous cover beneath *P. chilensis* could be due to the allopathic effect of its litter or roots. If the site is to be reforested with *V. seyal*, it should be combined with M*. denhamii* and/or, if *P. chilensis* is to be used, with *I. kotschyana* and/or *C. rotundus*. The mycorrhizal potential of these grasses would be a factor in the success of reforestation. The risk in these cases would be the loss of monocotyledons such as *E. tremula* and *E. prieuri*, which provide fodder for cattle, sheep, and goats in the area. Another risk associated with the use of *P. chilensis* is a loss of herbaceous diversity.

Estimation of MPNs of propagules capable of generating mycorrhizae shows that soils collected under the trees have a higher mycorrhizal infectivity potential. MPNs are higher in soils beneath *V. seyal* trees than in soils beneath *P. chilensis* trees. During the rainy season, a large number of herbaceous plants under the trees also contribute to increase root fragments in the soils and so increase the mycorrhizal infectivity potential. Soil MPN values decrease in soils outside the canopies of trees. This decrease may be related to the fact that soils outside the canopies of trees are predominantly occupied by the grasses *E. tremula* and *E. prieuri* which have low living root FM and IM. It is reported in the scientific literature that AMF are widely represented in saline soils (Aliasgharzadeh et al., 2001) and they can improve salt stress tolerance (Giri et al., 2007). However, salted soil can interfere with the establishment of a functional mycorrhizal symbiosis (Juniper and Abbott, 1993; Juniper and Abbott, 2006); this may justify the low values of MPN soils outside the canopies of trees. In contrast, the roots of herbaceous species such as *M. denhamii*, *D. hirtum*, *I. kotschyana*, and *Z. glochidiata*, which are predominantly present beneath *V. seyal* trees, and the roots of *V. seyal* trees have higher FM and IM and, thus, contribute significantly to increase MPN in soils under the trees. Their presence also supports the renewal of propagule stocks. The results confirm that *V. seyal* and *P*. *chilensis* trees form mycorrhizal symbioses with AMF. The works of Ingleby et al. (1997) in Senegal reported that 64% of *Prosopis juliflora* plants were colonized by AMF. Measurements of FM and IM show that the roots of *P. chilensis* and *V. seyal* plants have good mycorrhization potential.

FM and IM vary significantly between species (*V. seyal* or *P. chilensis*) when plants of these species are grown on the same soil types containing their own OM, respectively. FM and IM are significantly higher with *V. seyal* roots than with *P. chilensis* roots, respectively. The lower soil salinity beneath the trees of *V. seyal* could be a more favorable factor for the establishment of the AMF symbiosis than higher soil salinity beneath *P. chilensis*. The higher soil moisture beneath the trees of *V. seyal* could also be a more favorable factor compared with soils beneath *P. chilensis* where the salinity constraint is exerted on the AMF. When comparing FM and IM on soils under the canopies of trees and outside the canopies of trees, FM and IM are significantly higher for the roots of plants grown on soils beneath the trees.

In the presence of OM, root FM decreased for both *V. seyal* and *P. chilensis*. The roots of *V. seyal* plants have higher FM than those of *P. chilensis* regardless of the percentage of OM in the soil. In the presence of 30% OM, the FM of *V. seyal* roots remained high at more than 80% compared with *P. chilensis* roots which were at 52%. Thus, for *V. seyal*, leaf litter does not hinder the establishment of symbiosis with AMF, and even an OM amendment would be more favorable to plant growth. These results are in line with the work of Baar et al. (1994) and Conn and Dighton (2000) who similarly show that leaf litter extracts influence AMF growth. Increases in soil OM have a less repressive impact on *V. seyal* plants than on *P. chilensis* plants in terms of FM. The increase of soil OM content from 10% to 20% does not affect the FM of *V. seyal* roots, but it decreases significantly the FM of *P. chilensis* roots. For *V. seyal* roots, the increase of OM content to 50% induces a significant decrease of FM. However, a 30% increase in OM could be beneficial as it does not interfere with mycorrhization.

For *P. chilensis*, a low increase in soil OM content from 0% up to 10% does not induce a decrease on root FM. However, an increase of soil OM content from 10% to 20% decreases significantly the root FM. So, no significant decrease in root FM noted with OM levels from 20% to 30% also means that 30% of OM will induce a decrease of root FM as well as a 50% increase of soil OM content decreased significantly the root FM. The IM of the roots of both *V. seyal* and *P. chilensis* decrease if the soil OM content increases in all percentages of OM. IM are still higher for the roots of *V. seyal* than the roots of *P. chilensis* for all percentages of OM. For *V. seyal*, the increase in all percentages of OM induced decreases in IM except from 20% to 30%. The highest IM are for 10% of OM that allows 43.8% of IM. For *P. chilensis*, an increase from 10% or 20% up to 30% in OM reduced significantly the IM, but a gradual increase of 10% from each level did not induce a significant decrease of IM. FM exceed 90% in soils at 0 m under the canopies of trees. The IM reached 48% and 57%, respectively, in the soils beneath the trees of *V. seyal* and the trees of *P. chilensis*. FM and IM are higher in the roots of plants grown on soil 0 m under the trees than in the roots of plants grown on soil 20 m outside the canopies of trees. So, the presence of trees enhances the mycorrhizal potential of soils, and this confirms the results showing higher MPN values for soils from plant rhizospheres. These AMF beneath *V. seyal* may be recommended in programs to rehabilitate the mycorrhizal potential of these saline soils. These autochthonous strains could be very effective in terms of colonization rate and symbiotic efficiency compared with collection strains as shown in the work of Estrada et al. (Estrada et al., 2013b; Estrada et al., 2013a). It is known that the existence of stressful conditions encourages plants to establish mycorrhizal symbiosis. If the roots of plants grown in soil 20 m from the trunks of trees have lower FM and IM values, this means that AMF are not abundant outside the canopies of trees. This low mycorrhizal potential of soils outside the canopies of trees may be related to the higher salinity levels at these locations as confirmed by the EC and Na^+^ content values. The mycorrhizal potentials of soils then decrease from the trunks of trees to the uncovered area.

Natural living roots collected from herbaceous plants and trees in the site show a diversity of mycorrhization potential. The IM and the FM of herbs are improved beneath *V. seyal*, concordant with the density and diversity of herbaceous species under *V. seyal*, but this is not the same case beneath *P. chilensis* where there is no abundance and diversity of herbaceous species. The IM and the FM of the most overgrown herbaceous species are enhanced at the expense of the other species under *V. seyal*. Non-parametric median comparison tests applied to the FM and the IM allowed, in both cases, to identify groups. With the FM, two groups were defined. In the first group, the herbaceous species *D. hirtum*, *B. gayana*, *I. kotschyana*, *A. indica*, *C. rotundus*, and *M. denhamii* were identified with FM that are greater than or equal to 70%; they are all associated with *V. seyal* except for *B. gayana*. The second group consists of *E. tremula*, *Z. glochidiata*, *E. prieuri*, and *B. vermiculare* with FM greater than or equal to 40% and are associated with *P. chilensis*.

Three groups were defined with the comparisons of the median of IM. The first group contains *I. kotschyana* and *D. hirtum* with IM values between 29.4% and 30.7% and which are associated with *V. seyal*. These two herbaceous plants have a good contribution to the mycorrhizal potential of soils beneath *V. seyal* considering their natural FM and IM. The second group consists of *A. indica*, *M. denhamii*, and *Blainvillea gayana* with intensity values between 17.8% and 18.36%. The third group consists of *C. rotundus*, *Z. glochidiata*, *E. tremula*, *E. prieuri*, and *B. vermiculare* with IM values between 0.2% and 10.17% and is associated with *P. chilensis*.

The first two groups are more homogeneous in terms of IM values and the third group is more heterogeneous with lower IM values. Groups defined according to FM are more heterogeneous than groups defined according to IM. The higher natural living root mycorrhization rates of *V. seyal* compared with *P. chilensis* may also be a consequence of a higher number of plant-parasitic nematodes (results not shown here) beneath *P. chilensis* trees competing with AMF in root colonization. This has already been reported in scientific literature. Yang et al. (2014) reported the negative effects of nematodes on mycorrhizal colonization. Borowicz (2001) also reported that interactions of plant-parasitic nematodes with AMF affect plant health as they share the same roots of their host plants and can then be directly affected by competition with each other or indirectly by physiological and chemical changes in the plant. Similarly, plants infected with plant-parasitic nematodes are exposed to physiological and biochemical changes that can affect their interactions with AMF (Frew et al., 2018). These plant-parasitic nematodes are ubiquitous and represent an important component of ecological functioning (Kardol et al., 2010).

## Conclusion

The presence of *V. seyal* reduces salinity levels as shown by soil electrical conductivity and soil sodium content. It also improves soil fertility and mycorrhizal potential and increases herbaceous diversity, species richness, and density of individual herbs. These improvements decrease in the soils if the distance to the trunks of trees increases. The presence of *P. chilensis* increases soil salinity levels and reduces the density of herbaceous individuals. Results showed that salinity levels, indicated by electrical conductivity and Na^+^ content, were lower in soils beneath *V. seyal* than in soils beneath *P. chilensis* and in soil 20 m from the trunks of trees. Soil fertility parameters such as carbon, nitrogen, organic matter, available phosphorus, total phosphorus, potassium, calcium, and cation exchange capacity are higher in soils under the trees compared with uncovered soils. They are higher beneath *V. seyal*. These levels decrease in the soils if the distance to the trunks of trees increases.

The presence of trees has a positive impact on the number and diversity of herbaceous plants. Herbaceous species’ richness is higher under the trees than outside the canopies of trees and is higher beneath *V. seyal* than beneath *P. chilensis*. Herbaceous species’ diversity decreases with increasing distance from the trunks of trees. Dicotyledonous plants are more represented than monocotyledonous plants. The high density of *M. denhamii* individuals around the trunks of *V. seyal* decreases outside the canopies of trees. The density of *E. prieuri* and *E. tremula* is very low under the trees but increases in soil outside the canopies. One species, *S. obtusifolia*, was only found outside the canopies of trees. The species *A. indica*, *P. dilatatum*, *W. indica*, *L. senegalense*, and *B. ramosa* were only found under the trees and seem to be maintained because of the presence of tree species.

Soil mycorrhizal potentials are highest under the trees and decrease outside the canopies of trees. They are higher beneath *V. seyal*. AMF most probable numbers are higher in soils beneath *V. seyal* than in soils beneath *P. chilensis*. They are much higher in soils at 0 m compared with soils 20 m from the trunks of trees. They are very low in soils outside the canopies of trees. Mycorrhization rates (FM and IM) decrease when soil OM content increases. *Vachellia seyal* roots had higher mycorrhization rates than *P. chilensis* roots in all tested levels of OM. The average IM values of herbaceous natural living roots taken directly from outside the canopies of trees and under the trees are almost equal. However, FM values are higher under the trees and are roughly equal under both canopies of trees. The average mycorrhization rates of herbaceous plants that have the highest indexes of cover are higher beneath *V. seyal* and decrease outside the canopies of trees. They are almost equal beneath *P. chilensis* and outside the canopies of trees. The natural living root FM for herbaceous plants and trees present two distinct groups: one group with values greater than or equal to 70%, including *V. seyal*, and one group with values less than or equal to 40%, including *P. chilensis*. The IM values present three groups: one group with IM between 29.4% and 30.7%, a second group with IM between 17.8% and 18.36%, and a third group with IM between 0.2% and 10.17%. Our results indicate that *V. seyal* is a good species for the reclamation of saline lands.

## Data availability statement

The original contributions presented in the study are included in the article/supplementary material. Further inquiries can be directed to the corresponding authors.

## Author contributions

Mansour THIAO is the main author of the article, he designed and set up all the experiments. He followed the experiments, collected and analyzed the data and wrote the first manuscript. He also wrote the final paper integrating the suggestions of the other authors. Godar SENE and Moustapha NDIAYE contributed to the soil sampling and phytosociological studies during the phtytosociological surveys, the identification of species, the estimation of abundance-dominance coefficients, the collection of roots in situ and the transport of samples. Godar SENE contributed to the design of some of the experiments on soil microbial potential. He also contributed to the formatting of the manuscript. El Hadji Samba Ndao SYLLA supervised and followed the different steps of the design of the experiments and corrected the versions of the manuscript. All authors listed have made a substantial, direct, and intellectual contribution to the work and approved it for publication.
